# The integrative omics of white-rot fungus *Pycnoporus coccineus* reveals co-regulated CAZymes for orchestrated lignocellulose breakdown

**DOI:** 10.1371/journal.pone.0175528

**Published:** 2017-04-10

**Authors:** Shingo Miyauchi, David Navarro, Sacha Grisel, Didier Chevret, Jean-Guy Berrin, Marie-Noelle Rosso

**Affiliations:** 1 Aix-Marseille Université, INRA, UMR 1163, Biodiversité et Biotechnologie Fongiques, BBF, Marseille, France; 2 PAPPSO, Micalis Institute, INRA, AgroParisTech, Université Paris-Saclay, Jouy-en-Josas, France; USDA Forest Service, UNITED STATES

## Abstract

Innovative green technologies are of importance for converting plant wastes into renewable sources for materials, chemicals and energy. However, recycling agricultural and forestry wastes is a challenge. A solution may be found in the forest. Saprotrophic white-rot fungi are able to convert dead plants into consumable carbon sources. Specialized fungal enzymes can be utilized for breaking down hard plant biopolymers. Thus, understanding the enzymatic machineries of such fungi gives us hints for the efficient decomposition of plant materials. Using the saprotrophic white-rot fungus *Pycnoporus coccineus* as a fungal model, we examined the dynamics of transcriptomic and secretomic responses to different types of lignocellulosic substrates at two time points. Our integrative omics pipeline (SHIN+GO) enabled us to compress layers of biological information into simple heatmaps, allowing for visual inspection of the data. We identified co-regulated genes with corresponding co-secreted enzymes, and the biological roles were extrapolated with the enriched Carbohydrate-Active Enzyme (CAZymes) and functional annotations. We observed the fungal early responses for the degradation of lignocellulosic substrates including; 1) simultaneous expression of CAZy genes and secretion of the enzymes acting on diverse glycosidic bonds in cellulose, hemicelluloses and their side chains or lignin (i.e. hydrolases, esterases and oxido-reductases); 2) the key role of lytic polysaccharide monooxygenases (LPMO); 3) the early transcriptional regulation of lignin active peroxidases; 4) the induction of detoxification processes dealing with biomass-derived compounds; and 5) the frequent attachments of the carbohydrate binding module 1 (CBM1) to enzymes from the lignocellulose-responsive genes. Our omics combining methods and related biological findings may contribute to the knowledge of fungal systems biology and facilitate the optimization of fungal enzyme cocktails for various industrial applications.

## Introduction

The bioconversion of agricultural, forestry and industrial green wastes into renewable sources of energy with bio-based molecules is highly desirable. Innovations in ecologically-friendly technologies will lead to improvements in green chemistry and biorefinery, and contribute to the circular economy. Lignocellulose, the major component of plant biomass, is highly recalcitrant to enzymatic degradation because the cellulose forms crystalline structures and the lignin sets dense polyaromatic polymers. Great efforts have been made to convert lignocellulosic saccharides into biofuels, biogas, and bio-based chemicals [1,2]. Lignocellulose has enormous potential in the production of phenolics and aromatics-based products [3]. However, the problems are how to break down lignin unamenable to biochemical transformation and to degrade cellulose efficiently.

A solution to such technical challenges may be found in the nature. Some forest-dwelling mushrooms are evolved to break down plant materials and release (poly)saccharides and (poly)aromatics from lignocellulose. Saprotrophic white-rot fungi are highly specialized to decompose dead plants and convert them into consumable carbon sources [4]. Phylogenetic reconstructions showed that white-rot fungi evolutionarily gained specific groups of enzymes to break down recalcitrant lignocelluloses [5,6]. The gene repertoires of saprotrophic white-rot fungi are enriched in enzymes for oxidative degradation of lignocellulose, called auxiliary activity enzymes (AA) classified in the CAZy database [7]. There are different types of Carbohydrate Active Enzymes (CAZymes) in the CAZy database including glycoside hydrolases (GH), carbohydrate esterases (CE), polysaccharide lyases (PL), glycosyltransferases (GT), and carbohydrate binding modules (CBM) [8]. There are some known synergistic activities among the different families of CAZymes. For example, synergies between endoglucanases, cellobiohydrolases and beta-glucosidases result in an efficient cellulolytic enzyme system, which collective activity is higher than the sum of the activities of the individual enzymes [9]. Other synergies exist between oxido-reductases for the oxidative cleavage of lignocellulose. GMC oxidoreductases including cellobiose dehydrogenases (CDH; CAZy families AA3_1 and AA8) and AA3-2 donate electrons to lytic polysaccharide monooxygenases (LPMO, family AA9) for the oxidative cleavage of cellulose polymers [10–12]. Copper radical oxidases (CAZy family AA5) give hydrogen peroxide to manganese peroxidases (MnP; family AA2) for the oxidative cleavage of lignin [13]. There may be as-yet-to-be-known synergies between the fungal enzymes. Thus, it is necessary to explore transcriptomic and proteomic regulations of CAZymes.

Our general understanding of the regulation of fungal enzymes in lignocellulose deconstruction is limited. On one hand, ascomycetes fungi have been studied mainly for the regulation of genes coding for cellulases and hemicellulases [14]. On the other hand, studies of wood-decay basidiomycetes fungi have been focused on the role of oxidative enzymes and their interplay during lignocellulose breakdown [15–17]. Meanwhile, white-rot fungi from the genus *Pycnoporus* are known to degrade cellulose, hemicelluloses and lignin, and the versatility of the genus for biotechnological applications is recognized [18,19]. The *Pycnoporus* strain *P*. *coccineus* CIRM-BRFM310 was previously studied regarding the roles of cellulases, hemicellulases, esterases and lignin active peroxidases during cultivation on pine and aspen [16]. However, the enzymatic mechanisms for the alleviation of plant biomass recalcitrance by this fungus remained to be elucidated.

Fungal responses to the source of carbon are dynamic. Fungi utilize specific groups of genes to execute lignocellulose breakdown, metabolization, and detoxification of molecules during the degradation process. Such orchestrated molecular machineries are required to convert plant biomass into a carbon source. Time-course investigations of fungal gene co-regulations and corresponding protein co-secretions induced by plant biomass are well suited to examine the systematic fungal depolymerization of lignocellulose. However, genome-wide transcriptomic and secretomic activities are complex. Capturing just a single time point of fungal transcriptomic activity involves over ten thousands genes showing various transcription levels. The number of observations increases exponentially when we add the number of biological replicates, different growth conditions, and time points. The addition of secretomic information gives an extra layer of complexity.

To extract biologically meaningful patterns from such high-dimensional omics data, we have developed the multi-omics profiling platform, Self-organizing map Harboring Informative Nodes with Gene Ontology (SHIN+GO). Genome-wide omics models constructed with the platform are designed to pinpoint biological activities of interest that would otherwise be buried in the high-dimensional data. A prototype of the platform was previously developed as Applied Biomass Conversion Design for Efficient Fungal Green Technology (ABCDEFGT), which had a limited capacity of processing large-scale omics data [20]. As one of the key components of this platform, Self-organizing map (SOM) is an algorithm constructing a neural network with given input data in an unsupervised manner [21]. SOM reduces the number of features in high-dimensional data by grouping similar items and forming clusters. It has a unique property of making two-dimensional maps suitable for large-scale data visualization. The method has been used to generate neural networks of genome-wide genes and identify condition-specific responses in transcriptomes of newly sequenced three fungal species with limited gene annotations [20].

In this study, we used the improved platform (SHIN+GO) to generate dynamic genome-wide integrative omics models with two time points. We profiled the early transcriptomic and secretomic responses of *P*. *coccineus* CIRM-BRFM310 at day 3 and 7. The fungal strain was grown with three types of recalcitrant lignocellulosic substrates (i.e. cereal straw, softwood, and hardwood) to capture an overview of the fungal adaptive responses to plant biomasses with different compositions of cellulose, hemicelluloses, and lignin. The selected plant substrates are considered as sustainable resources for green technologies [1,22]. We built integrative omics models in an unsupervized machine learning manner and used them as a guide to explore biologically interesting parts of the large-scale omics data (i.e. omics hotspots). We interpreted the findings in terms of fungal transcriptomic, secretomic, and metabolomic responses to the lignocellulosic substrates.

## Results and discussion

### Fungal omics models for the lignocellulosic substrates

We investigated co-regulated genes with corresponding co-secreted proteins of *P*. *coccineus* CIRM-BRFM 310 growing on four different substrates at day 3 and day 7 in order to see the early transcriptomic and secretomic adaptations. Maltose, which is an easily assimilated source of carbon, was used as a control, and ground wheat straw, pine wood, and aspen wood were used as models for gramineae, softwood, and hardwood biomass, respectively.

Under the conditions imposed in this study, optimal fungal growth was observed at day 3 on lignocellulosic substrates whereas a lag phase of 72 hours was observed on maltose (S1 Fig). A time-course experiment measuring the quantity of maltose consumed by the strain showed the maltose initially added to the basal medium was completely depleted in the culture medium at the point of day 3 (Miyauchi et al. in prep). Therefore, the fungal responses for the lignocellulosic substrates at day 3 and 7 were not affected with the residual maltose. The normalized read counts obtained from the RNA-seq of three biological replicates grown under each of the four conditions at each time point were highly correlated (> 0.9 correlation co-efficient; S2 Fig), and the distributions of the normalized read counts from the replicates were also almost identical (S3 Fig). A total of 26 housekeeping genes showed a stable transcript level between the two time points, suggesting that the physiological state of *P*. *coccineus* was comparable between all cultivation conditions (S4 Fig). Thus, we concluded that the obtained biological data were consistent and comparable to each other.

**Fig 1:**
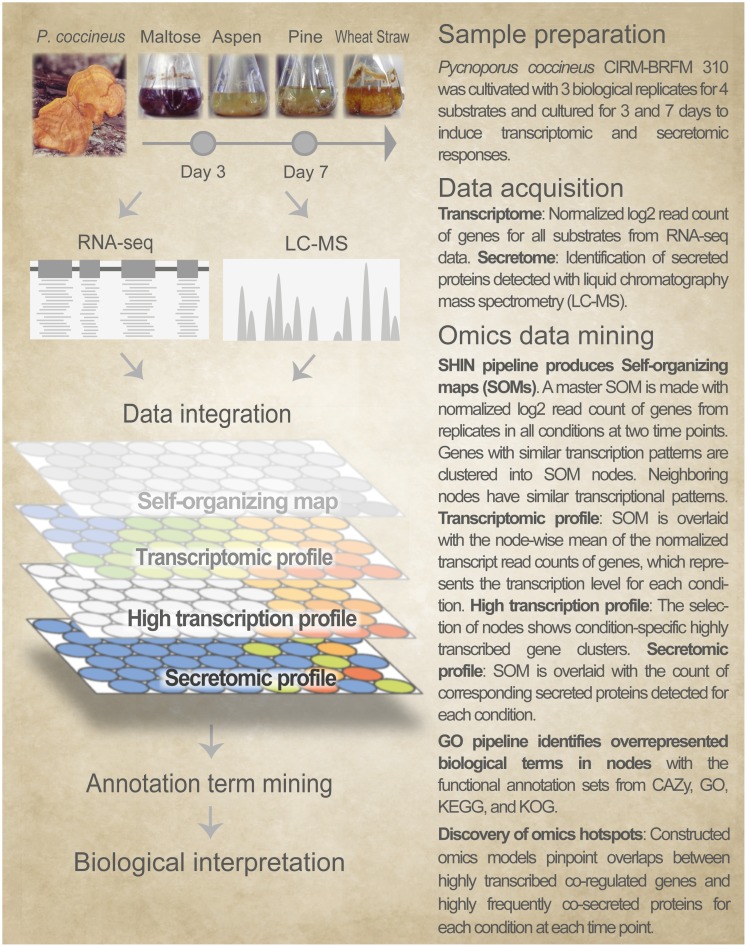
Overview of the genome-wide integrative omics profiling of *Pycnoporus coccineus* CIRM-BRFM 310 at two time points. The SHIN+GO platform; 1) integrated the fungal transcriptome from RNA-seq data and the secretome from liquid chromatography mass spectrometry; and 2) assisted the biological interpretation of the outputs of the omics models with functional gene annotations.

We generated fungal omics models with transcriptional changes at two time points. The models constructed in this study were more vast and congruent than those built with a single time point in the previous study [20]. A total of 274,320 points of observations combined from two time points (i.e. 4 conditions x 3 replicates x 2 time points x 11,430 genes with corresponding secretomes) were taken into account to construct omics models of *P*. *coccineus*. We used a multi-omics profiling platform, Self-organizing map Harboring Informative Nodes with Gene Ontology (SHIN+GO; Fig 1).

The first part of the SHIN+GO platform, Self-organizing map Harboring Informative Nodes (SHIN; the improved version of ABCDEFGT; Miyauchi et al., 2016), was used to merge genome-wide omics data. The SHIN pipeline constructed a single master self-organizing map (SOM) with the normalized read counts of 11,430 genes from the triplicates grown under the four cultivation conditions at day 3 and 7. The genes were sorted into 456 nodes according to their similar transcriptomic patterns. Next, the count of secreted proteins was overlaid onto the master SOM. As a result of this integration of data, SHIN provides nodes made of clustered co-regulated genes (transcriptomes) with corresponding co-secreted proteins (secretomes). The second part of the SHIN+GO platform, Gene Ontology (GO), was newly developed for this study; 1) to measure the frequency of gene functional annotations present in the nodes; and 2) to biologically interpret the outputs of the genome-wide omics models generated. Biological terms with statistically enriched occurrence in a node were used as an indicator of biological functions for the grouped genes and proteins.

**Fig 2:**
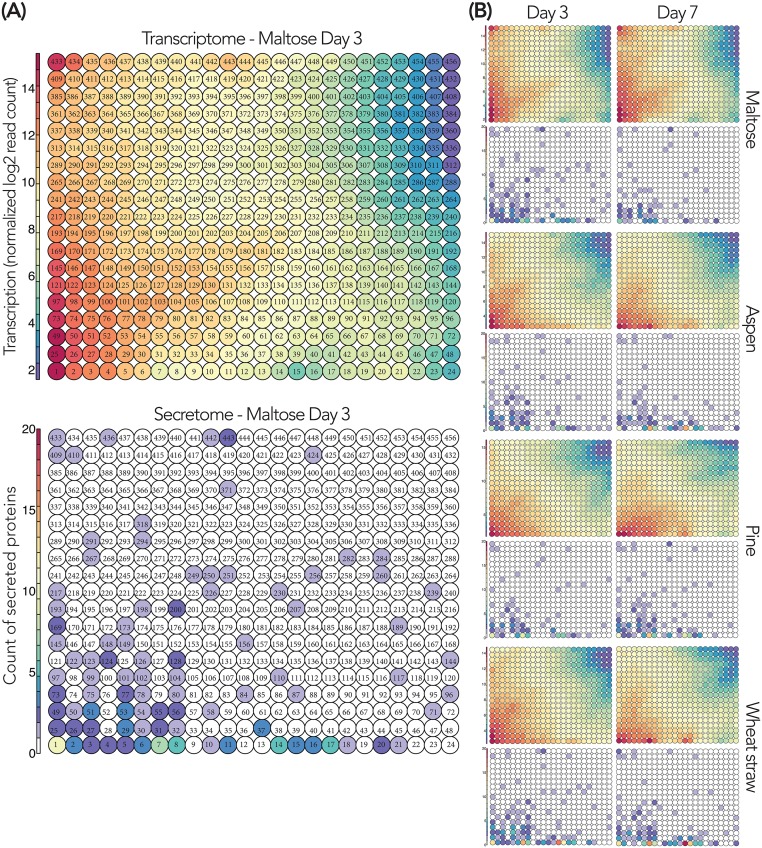
Genome-wide integrative omics models of *P*. *coccineus* in response to the substrates at day 3 and 7. Transcriptomic topography: Mean transcription levels per node for each cultivation condition. Secretomic topography: The total count of secreted proteins per node indicates secretion hotspots. (A): Magnified version of the topographies. The node identification is labeled (i.e. 1 to 456). (B): Transcriptomic and secretomic topographies from the four substrates. An animated version of transcriptomic topographies is available (S1 File).

All transcriptomic and secretomic topographies are comparable as the positions of the nodes are fixed in the maps (Fig 2A), enabling the instant visual inspection of omics hotspots where biologically intensive activities can be observed (Fig 2 and S5 Fig). The node-wise mean of the normalized transcript read counts reflects the transcription level in response to each condition. The secretomic topography represents the count of secreted proteins detected from the culture medium. Some nodes contained high counts of secreted proteins, indicating hotspots of co-secreted proteins.

Globally, there is a consistency that the transcriptomic topographies showing high transcription contain higher counts of secreted proteins in the corresponding secretomic topographies. It suggests that the high transcription of genes generally leads to the secretion of their proteins (Fig 2). Another observation is that the transcriptomic and secretomic patterns resulting from cultivation on different substrates were slightly different from each other. Particularly, the patterns obtained from maltose differed from those from the lignocellulosic substrates (aspen, pine, wheat straw). Notably, there are overlaps between some of the nodes showing high transcription and intensive secretomic responses to the lignocellulosic substrates (i.e. nodes 1 to 17). We considered such nodes as ‘omics hotspots’ involved in biologically-interesting events at the transcriptomic and secretomic levels. We found that several of these nodes were enriched in CAZyme coding genes (discussed in later sections).

**Fig 3:**
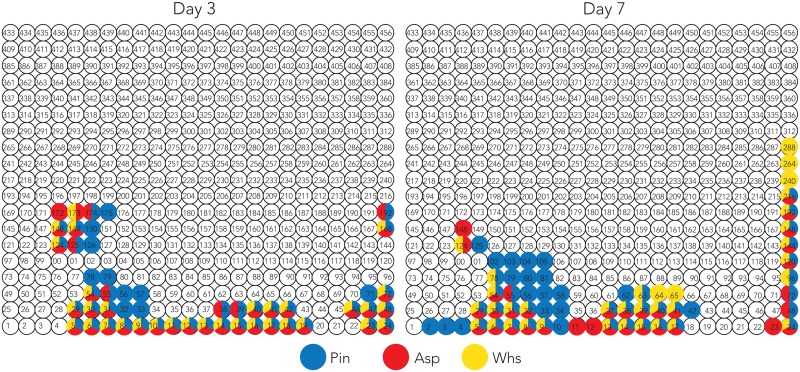
Transcriptomic changes of the lignocellulosic substrate-specific nodes from day 3 to day 7. The specific transcription patterns for the lignocellulosic substrates were extracted from Fig 2. The highlighted nodes met either of two criteria; 1) > 11.7 log2 read counts; or 2) > 2 log2 fold changes on aspen (Asp), pine (Pin), and wheat straw (Whs) in comparison with maltose at each time point.

Nodes showing up-regulation and high transcription for the lignocellulosic substrates were determined from the omics models. Nodes that met either of two following criteria were selected for the further analysis; 1) > 11.7 log2 transcript read counts, which constitute above 90th percentile of the transcription level of the entire gene group; 2) > 2 log2 transcriptional changes compared to maltose at each time point (Fig 3).

In the transcriptome at day 3, a majority of genes in the nodes showed a similar response to the three lignocellulosic substrates (i.e. 32 nodes showed high transcription or high up-regulation on each of aspen, pine, and wheat straw; Fig 3). There were eleven nodes showing substrate-specific responses to either only pine (ten nodes) or aspen (one node). However, there were no nodes specific to wheat straw. At day 7, a higher number of genes in the nodes were highly transcribed or highly up-regulated on a single substrate compared to day 3 (i.e. nineteen, four, and five nodes for pine, aspen, and wheat straw respectively). The observation indicated that common molecular functions were triggered by the exposure to the lignocellulosic substrates at the early stage (day 3). Then, the fungus adjusted more specific responses to the specific substrate over time at the later stage (day 7). Remarkably, the number of pine-specific nodes was increased by nine from day 3 (nodes 32, *33, *56, *57, 71, 78, 79, *126, 150, 175) to day 7 (*2, *3, *4, 10, *33, 34, 42, *56, *57, *58, 62, 79, *80, 81, *102, 103, 104, 105, *125). Four and ten of these nodes at day 3 and day 7 respectively contained statistically enriched gene annotations (i.e. the nodes with asterisks above and discussed in later sections).

### Transcriptomic responses to the lignocellulosic substrates

We attempted to interpret the fungal adaptations to the lignocellulosic substrates at the transcriptomic level using the enriched gene annotations present in the nodes. The frequency of gene annotations in the 456 nodes of the omics models revealed that five nodes (1, 7, 8, 14, 15) contained a significant number of genes coding for CAZymes (S1 Table and S4 File). Among them, four nodes (7, 8, 14, 15) showed high transcription during the growth on the lignocellulosic substrates compared to maltose (Fig 3 and Table 1). These nodes contained the highest number of frequently secreted proteins (secretomic topographies in Fig 2), suggesting that the corresponding CAZymes were contributing to the decomposition of the lignocellulosic substrates. Nodes in proximity share similar transcriptomic patterns due to the nature of SOM used for the construction of the omics models [20]. Hence, the regulations of genes coding for CAZymes in nodes 7 and 8 on the one hand, 14 and 15 on the other hand can be considered as co-regulated groups.

**Table 1:** Nodes with highly enriched CAZyme coding genes up-regulated on the lignocellulosic substrates.

Node	CAZymes
7	AA2, AA3_3, AA8-AA3_1, AA9, AA9-CBM1(2), CBM1-CE16, GH1, CBM1-GH10, CBM1-GH5_7, CBM1-GH6, GH7, GH51, GH16, GH30, GH35, GH74-CBM1, GH79, PL8_4
8	AA3_2(2), AA5_1, CE4, GH3, GH28, GH55, GH76, GH92
14	CBM1-CE1(2), CE8, CBM1-CE15, CE16, GH3, CBM1-GH10, CBM1-GH5_5, GH12(2), GH131-CBM1, GH28, GH32, GH43(2), GH45, GH53, GH78, GH115
15	AA2, AA9(5), CE16, GH28(2), CBM1-GH5_5, CBM1, GH7(2)

Different types of CAZymes present in node 7, 8, 14, and 15. AA: Auxiliary activity enzymes, GH: Glycoside hydrolases, CE: Carbohydrate esterases, CBM1: Carbohydrate binding module 1 (CBM1) [8]. The number of gene copies is indicated in brackets.

Each of four nodes contained a set of CAZymes active on the four types of lignocellulose polymers (i.e. cellulose, hemicelluloses, pectin and lignin), suggesting that the fungus simultaneously recruited a variety of enzymes and executed the orchestrated depolymerization (Table 1). There were eight LPMO (AA9) coding genes in nodes 7 and 15 that were up-regulated in response to lignocellulosic substrates at the two time points (Table 1). Other highly up-regulated genes were two predicted glucose-methanol-choline (GMC) oxidoreductases (family AA3_2; protein ID: 1463000, 1465734) and one glyoxal oxidase (AA5_1; 1480943) in node 8, which could give hydrogen peroxide to AA2 in nodes 7 (1468611) and 15 (1430659) for their enzymatic activities (Table 1). There were three LPMOs in node 7 (AA9; 1374028, 1382161, 1428145) along with the presence of one cellobiose dehydrogenase (AA8-AA3_1; 1401955) and two GMC oxidoreductases (AA3_2; 1463000, 1465734) in node 7 and 8, suggesting co-regulation for the oxidative cleavage of cellulose polymers [12]. Also, there were genes encoding hemicellulose-active enzymes such as glycosyl hydrolases (GH) and carbohydrate esterases (CE). Some of the GHs are for the degradation of xylan. GH10 acts on the main chain of xylan, while GH43 and 51 are arabinofuranosidases for the arabinose substitution of xylan. GH115 is a glucuronidase for the glucuronoyl substitution of woody xylan. Meanwhile, some of the CEs are targeting acetyl xylans (CE4, CE16) and 4-O-Methyl glucuronoyl side chains (CE15). In addition, nodes 7 and 14 were highly enriched with CBM1 associated CAZymes (Table 1 and S2 File; adjusted p < 0.01). CBM1 has an affinity to crystalline cellulose and directs CBM1 associated enzymes to potentiate cellulolytic activities on insoluble substrates [23,24].

**Fig 4:**
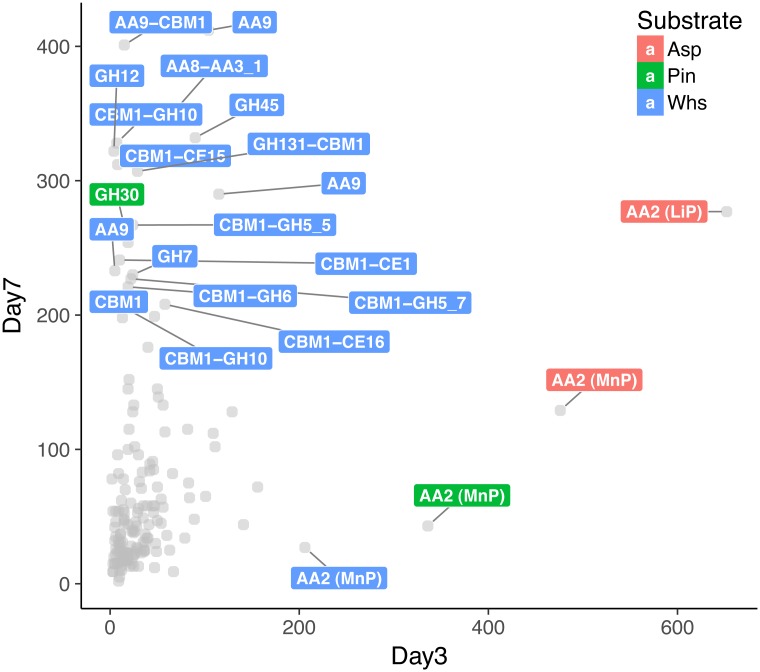
Transcription intensity of CAZyme coding genes in nodes 7, 8, 14, and 15 at day 3 and day 7. The X and Y axes represent the values of transcription induction factor (TIF). TIF values > 200 are labeled at each time point. TIF were estimated by squaring log2 fold change values of the transcript read counts on lignocellulosic substrates compared to the control condition with maltose. Detailed information is provided (S2 Table).

We visualized the transcription intensity of CAZyme-coding genes from nodes 7, 8, 14, and 15 in order to capture the specific regulation patterns at two time points (Fig 4). Globally, the transcript level of the genes for plant cell wall-active enzymes went up from day 3 to day 7.

At day 3, the most highly up-regulated CAZymes were the lignin-active peroxidases in nodes 7 and 15 (AA2-Mnp/Lip; protein ID: 1468611, 1431101), which were less-strongly up-regulated at day 7, suggesting that their regulations were finely controlled. The tight regulation could possibly expose cellulose and hemicellulose polymers from lignin while minimizing oxidative damage to the fungus. This phenomenon is in accordance with the dynamics of MnP or LiP gene transcription in *P*. *carnosa* and the detection of the enzymes in the secretomes of *C*. *subvermispora* [25–27]. At day 7, almost all of highly up-regulated CAZymes (transcription induction factor > 200) were from the cultivation on the wheat straw substrate (Fig 4). There were 18 CAZymes and the majority had a CBM1 attached including four AA9, AA8-AA3_1, two GH10, GH5_5, GH5_7, GH6, GH7, GH12, GH45, GH131, CE1, CE15, and CBM1. The cellobiose dehydrogenase-coding gene (CDH; AA8-AA3_1; protein ID:1401955) co-regulated with three LPMOs (AA9) grouped in node 7 (Table 1) showed strong up-regulation on the three lignocellulosic substrates (e.g. transcription induction factor 351 on the wheat straw substrate at day 7; Fig 4), suggesting that the CDH might promote the activity of these LPMOs via electron transfer [28]. LPMO coding genes were seemingly co-regulated with hydrolytic enzymes active on beta-1,4-glycans such as GH3, GH5, GH6, GH7, GH45, and GH131. Since LPMOs display oxidative cleavage of crystalline cellulose [28], the combinations of these CAZymes indicated the simultaneous induction of hydrolytic and oxidative activities (Fig 4). Some LPMOs were highly up-regulated as the transcription induction factors were 115 at day 3 and 412 at day 7 on the wheat straw substrate (AA9; 1374028, 1417214; Fig 4 and S2 Table). LPMOs in different fungal species had similar transcriptional regulations during lignocellulosic degradation. The white-rot fungi *Phlebia radiata* and *Phlebiopsis gigantea* showed up-regulation of the genes during the growth on spruce and loblolly pine wood respectively [29,30]. Also, secreted LPMOs were found in *Phanerochaete chrysosporium* and *Ceriporiopsis subvermispora* [26,27,31,32].

Incidentally, expansin (EXPN) showed unique transcriptional patterns for the lignocellulosic substrates in comparison to maltose (S4 File). While four expansin coding genes showed the moderate up-regulation only on the pine substrates at day 3 and 7 (EXPN; 1451679, 1467568, 1470236, 316489), other ten genes were mostly down-regulated on each of the lignocellulosic substrates at two time points (EXPN; 1440495, 1439344, 1463757, 1477520, 1424314, 1422224, 1463980, 1386069, 1373750, 148891). There might have been some transcriptional regulations induced by the pine substrate. This peculiar phenomenon remains to be elucidated.

### Secretomic responses to the lignocellulosic substrates

We examined secretomic responses to the lignocellulosic substrates by comparing secreted proteins detected at day 3 and day 7. The CAZyme-enriched nodes (7, 8, 14, 15) were part of six nodes that showed the highest number of secreted proteins (> 9 protein count) among the total of 49 nodes containing secreted proteins (Table 2). A greater number of proteins was detected at day 7 compared to day 3, suggesting the accumulation of secreted enzymes in the culture medium over time.

**Table 2:** Secretomic responses to the lignocellulosic substrates at day 3 and day 7.

Node	Day 3 only	Day 3 and Day 7	Day 7 only
7		AA8-AA3_1, AA9-CBM1, AA9, CBM1-CE16, CBM1-GH10, CBM1-GH5_7, GH79, Peptidase S53	AA9-CBM1
8	Ceramidases	GH28	AA3_2, AA5_1
14		Aldose_1-epimerase, CBM1-CE1 (2), CBM1-CE15, CBM1-GH10, CBM1-GH5_5, CE16, CE8, GH12 (2), GH131-CBM1, GH43, GH45	GH53, GH78, GH115
15		AA9 (4), Carboxylesterase_and_related_proteins (#1468641), CBM1-GH5_5, GH7 (2), SSP (#1437297), Unknown (# 1439153)	AA2 (LiP), CE16, Unknown (#1446065)

Secreted proteins were detected under the cultivations on the lignocellulosic substrates. Unknown: Proteins with unknown function. SSP: Small secreted proteins which size is smaller than 300 aa. #: Protein ID. The number of gene copies is indicated in brackets. Details of the selected six nodes are provided (S3 File).

Notably, the oxidative enzymes (AA2-LiP; protein ID: 1431101) for lignin degradation and the partner enzymes glyoxal oxidase (AA5_1, 1480943) and GMC oxido-reductases (AA3_2, 1463000, 1465734) for hydrogen peroxide production were detected only at day 7, suggesting that the secretion of such enzymes at day 3 could have been limited by post-transcriptional regulations. We evaluated the correlation between the transcriptome and secretome in response to each substrate at each time point. The two omics patterns were moderately correlated (the correlation co-efficient ranging from 0.30 to 0.44; p < 0.001; S3 Table). The moderate correlation could be due to the narrow window of our observation points (at day 3 and 7). Also, difficulties in the detection of proteins in the liquid fraction of culture media could have arisen from; 1) delays in mRNA translation; 2) fungal proteins bound to the substrates; or 3) a lower amount of proteins secreted than the detection limit.

### Metabolic adaptations to the lignocellulosic substrates

We investigated fungal metabolic adaptations during the growth on the lignocellulosic substrates at day 3 and day 7 using the annotation databases Gene Ontology (GO), Kyoto Encyclopedia of Genes and Genomes (KEGG), and EuKaryotic Orthologous Groups (KOG) [33–35]. The functional annotations found in all 456 nodes are provided (S4 File).

A total of 150 out of 456 nodes contained statistically enriched functional annotations (adjusted p < 0.01; S1 Table). There were twenty nodes showing either up-regulation/high transcription on two or three lignocellulosic substrates, and also up-regulation/high transcription specific to the pine substrate (S4 and S5 Tables).

The terms relating to putative hydrolases active on carbohydrates were enriched in nodes 7, 14, and 15 (S4 Table). Putative dehydrogenases, oxidoreductases or peroxidases were also enriched in nodes 7, 8, 19, 22, and 23. These enriched terms confirmed that the fungus executed hydrolytic and oxidative processes for the breakdown of the lignocellulosic substrates, which was discussed earlier in the transcriptomic and secretomic responses (Tables 1 and 2). The up-regulation of the genes was accompanied by predicted transporters in nodes 23 and 39 potentially involved in carbohydrate assimilation (S4 Table). The terms for predicted cytochrome P450s were enriched in nodes 9, 40, and 63, suggesting that the fungus adjusted to exposure to toxic compounds released from the lignocellulosic substrates during the degradation process (S4 Table). The findings suggest that the white-rot fungus *P*. *coccineus* might have used a common set of P450 genes for the fungal adaptation to diverse lignocellulosic substrates. The detoxification response of *P*. *coccineus* to lignocellulosic substrates somewhat differs from that of the brown-rot fungus *Postia placenta*, which showed a higher transcription of P450 genes and specific P450 protein secretion during the growth on pine (i.e. *Pinus strobes*) in comparison to aspen (i.e. *Populus gigantea*) [32]. The different culture conditions and compositions in wood extractives might have contributed to the variations of the fungal responses.

Nodes 2 and 3 were enriched with the terms for putative chitinases and putative 1,3-beta-glucan synthases respectively (S5 Table). Comparing the enriched terms with the CAZy annotations confirmed the predicted functions for one chitinase (protein ID: 1433997, CAZyme: GH18), with a chitin synthase (1467191, GT2) in node 2 and two b-1,3-glucan synthases (1436152 and 1479981, GT48) in node 3. These terms indicate that there might have been remodeling of the fungal cell wall during the growth on the pine substrate. In addition, four nodes (4, 33, 56, 58) had enriched terms for putatively involved in signal transduction such as protein kinases, which might have contributed to the signaling cascade driving the fungal response to the pine substrate (S5 Table).

## Conclusions

We examined transcriptomic and secretomic models of *P*. *coccineus* CIRM-BRFM 310 at two time points in response to three different types of lignocellulosic substrates representing gramineae, softwood and hardwood. The transcriptomic approach unveiled the dynamic changes of genome-wide transcription levels while the secretomic approach identified sets of enzymes involved in the synergistic depolymerization of the lignocellulosic substrates. Co-regulated genes and corresponding co-secreted proteins were determined and their biological roles were extrapolated using the enriched CAZymes and other functional annotations. Although our observations were limited to two time points, the fungus seemed to execute an almost simultaneous recruitment of various enzymes targeting cellulose, hemicelluloses, lignin and pectin, rather than a step-by-step degradation of the plant polymers. A composite of hydrolases, esterases and oxido-reductases active on plant cell walls might have been simultaneously secreted to achieve lignocellulose decomposition. Genes coding for enzymes associated with a carbohydrate binding module (CBM1) were strongly up-regulated and their proteins were secreted in response to the lignocellulosic substrates. Secreting abundant sets of CBM1-associated CAZymes could improve the efficiency of the degradation of plant cell wall polymers as CBM1 enhances the binding of CAZymes to cellulose. The plant cell wall-degrading CAZymes exhibited a global increase at the transcription level from day 3 to day 7, except for lignin active peroxidases (AA2), which displayed a unique early regulation at day 3. A large number of genes coding for LPMOs (AA9) appeared in the omics hotspots of our models, indicating a close involvement of this enzyme family for adaptive responses of *P*. *coccineus* to the complex substrates. Our integrative omics strategies enabled us to combine layers of biological information for better understandings of fungal machineries for decomposition of plant cell walls. The SHIN+GO platform is versatile and can be applied for comparative transcriptomics of different strains or species. Our findings may shed some light on the design of customized enzyme cocktails for the conversion of diverse and highly recalcitrant biomass resources.

## Materials and methods

### Fungal strains and cultures

*P*. *coccineus* CIRM-BRFM 310 was obtained from the International Center of Microbial Resources (CIRM-CF; https://www6.inra.fr/cirm_eng). 5mm discs of mycelium grown on malt agar were used to inoculate Roux flasks containing 200 ml of medium (five discs per flask) and incubated at 30°C. After 15 days, the mycelium was ground (ultraturax 10000 rpm, 60 s) in 100 ml of purified water (MilliQ, Millipore). Five milliliters of this suspension were used for the inoculation of each 250-ml Erlenmeyer flask containing 100 ml of basal medium: diammonium tartrate (1.84 g.L^−1^), Yeast Extract (2.5 g.L^−1^), Maltose (2.5 g.L^−1^), KH_2_PO4 (0.2 g.L^−1^), CaCl_2_·2H_2_O (1.32.10^−2^ g.L^−1^), MgSO4·7H_2_O (0.5 g.L^−1^), FeSO4·7H_2_O (0.07 g.L^−1^), ZnSO4·7H_2_O (7.77.10^−3^ g.L^−1^), MnSO4·H_2_O (3.63.10^−3^ g.L^−1^), CuSO4·5H_2_O (7.2.10^−4^ g.L^−1^) and thiamine (250.10^−3^ g.L^−1^). The test conditions were created by adding one of the following additional components: maltose (20 g.L^−1^ final), ground and sifted wheat straw < 2 mm (15 g.L^−1^), ground and sifted *Pinus halepensis* wood fragments < 2 mm (15 g.L^−1^) or 1 mm Wiley-milled *Populus grandidentata* (15 g.L^−1^) kindly provided by Dan Cullen (Forest Product Laboratory, USDA, Madison, WI). Incubation was carried out at 30°C in a rotary shaker (Infors AG) at 120 rpm. Assays were performed in triplicate.

### RNA preparation and RNA-sequencing

Total RNA was extracted from 3 day and 7 day-old cultures as described [16]. In short, mycelia were harvested by filtering through Miracloth (Calbiochem), squeeze dried, snap frozen in liquid nitrogen and stored at -80°C until use. Frozen fungal pellets were ground using the SamplePrep 6770 FreezerMill (Spex). RNA was extracted from one hundred milligram of ground mycelium in 1 mL TRIZOL (Ambion). RNAs were precipitated with isopropanol (Sigma-Aldrich), treated with DNAse I (QIAGEN) and resuspended in 25 μL RNAse Free water. RNA purity and integrity were analyzed on NanoDrop Spectrophotometer and Agilent 2100 BioAnalyzer. For RNASeq, cDNA libraries were prepared using the TrueSeq RNA-Seq Sample Prep Kit V2 (Illumina Inc., San Diego, CA), and submitted to sequencing using Illumina 2x75 bp technology (Beckman Coulter Genomics). The RNA-seq data are available on NCBI's Gene Expression Omnibus and accession number GSE94878 [36].

### Protein extraction and detection

Secreted proteins were collected from the same cultures, dia-filtered and identified by ESI-MS/MS as described in Couturier et al. (2015). Briefly, short SDS-PAGE runs were performed, allowing 10 μg of proteins to migrate on a 0.5 cm length. Each one-dimensional electrophoresis lane was cut into two slices of gel and protein identification was performed using PAPPSO “Plate-forme d'Analyse Protéomique de Paris Sud-Ouest” platform facilities. In-gel digestion was carried out according to a standard trypsinolysis protocol. Online analysis of peptides was performed with a Q-exactive mass spectrometer (Thermo Fisher Scientific), using a nanoelectrospray ion source. Protein identification was performed by querying MS/MS data against the genome *P*. *coccineus* BRFM 310 v1.0, together with an in-house contaminant database, using the X!Tandem software (X!Tandem Cyclone, Jouy en Josas, France). All peptides matched with an E-value lower than 0.05 were parsed with X!Tandem pipeline software. Proteins identified with at least two unique peptides and a log (E-value) lower than -2.6 were validated. The secretomic information is provided (S5 File).

### Genome and gene annotations

Functional annotations of *P*. *coccineus* with GO, KEGG, KOG, SignalP were obtained from Mycocosm, JGI (http://genome.jgi.doe.gov/Pycco1/Pycco1.home.html). CAZy annotations were obtained from the CAZy group led by Bernard Henrissat (AFMB, Aix Marseille University).

### Data preparation and manipulations

QCed reads from each library were aligned to the genome *P*. *coccineus* BRFM 310 v1.0 using TopHat [37] with only unique mapping allowed. R was used for data manipulations using our customized scripts [38]. Raw read counts obtained from HTSeq were used for the calculation using DESeq2 [39]. A total of 11,430 genes having more than five reads were selected for the analysis. The normalized log2 transformed read count of genes showed similar mean and distribution patterns. The log2 fold difference of the gene expression induced by cultivation on aspen, wheat straw, pine, compared to maltose, was calculated at each time point. The statistically significant genes were selected based on adjusted p value < 0.05 (FDR and Bonferroni correction). Normalized read counts of the genes were produced with the function, counts in DESeq2, which were subsequently log2 transformed [39].

### Transcript abundance of housekeeping genes

Housekeeping genes coding for chitin synthases and NADH dehydrogenases were chosen for comparison of transcription levels between day 3 and 7. Normalized log2 read counts were compared for a total of 26 genes between the combined triplicates from each cultivation condition.

### Construction of integrated omics models

Two-time-point transcriptome and secretome models were constructed using the SHIN pipeline (i.e. the ABCDEFGT workflow with improved capacity) [20]. A SOM was trained with the normalised read count of all conditions using Rsomoclu [40] on the computer clusters at Mesocentre (https://equipex-mesocentre.univ-amu.fr/en/welcome-mesocentre-aix-marseille-universite/). The matrix of 24 x 19 (456) was used with four neighbouring nodes. The resolution of 25 genes per node was used, which was empirically determined. The epoch of 1000 times more than the map size was applied, i.e. 456,000 = 456 (map size) multiplied by 1000. The initial radius for SOM calculation was determined using neighbour distance function in R Kohonen package [41]. The following outputs were visualized; 1) transcriptomic models of all biological replicates in four cultivation conditions at two time points; and 2) combined transcriptomic and secretomic models in four cultivation conditions at two time points.

### Determination of time-course secretomic profiles of *P*. *coccineus*

Nodes containing high numbers of secreted proteins were determined from the 3- and 7-day cultivation on the aspen, pine, and wheat straw substrates. Protein IDs detected in the cultivations on maltose were removed as a background. There were 49 nodes containing secreted proteins for the lignocellulosic substrates. Then, a total of 6 nodes were selected based on the total occurrence of proteins detected above 90th percentile of the protein counts in 49 nodes.

### Correlation of transcriptome and secretome

Spearman's rank correlation was calculated for; 1) genome-wide transcriptome and secretome; and 2) selected genes for only secreted proteins. The correlation coefficients were calculated with the mean transcription level of all nodes from the normalised log2 reads (transcriptome) and the frequency of proteins secreted in each condition at each time point (secretome).

### Term-mining and enrichment analysis

The GO pipeline was used to count the frequency of gene annotations per nodes in the integrated omics models generated from the SHIN pipeline. It was performed using the individual annotation data sets CAZy database [7,8], The Gene Ontology (GO), Kyoto Encyclopedia of Genes and Genomes (KEGG), and EuKaryotic Orthologous Groups (KOG) [33–35]. The appearance of terms was counted using tm package [42]. The genes with the same annotation appearing more than twice in a single node were selected for enrichment analysis. P values of enriched gene annotations per node were calculated using a function phyper in R stats package for the hypergenometric test [43]. The following parameters were considered to estimate the overrepresentation (enrichment) of biological terms in nodes; 1) the number of specific terms of interest in nodes; 2) the number of all terms present in the nodes; 3) the number of specific terms of interest in the genome; 4) the number of all terms present in the genome excluding the specific terms of interest. P values were adjusted using Benjamini-Hochberg (FDR). Annotations with adjusted p values of FDR < 0.01 were considered to be statistically significant.

## Supporting information

S1 Fig Growth of *P*. *coccineus* CIRM-BRFM310 on the substrates. (PDF)

S2 Fig Correlation of normalized read counts from the three biological replicates in four conditions at two time points. (PDF)

S3 Fig Box and density plot of the normalized log2 read counts from three biological replicates in four cultivation conditions at two time points. (PDF)

S4 Fig The normalized log2 transformed read count of housekeeping genes. (PDF)

S5 Fig Transcriptomic profiles of three biological replicates in four cultivation conditions at two time points. (PDF)

S1 Table Statistically significant enrichments in gene annotations per node. (PDF)

S2 Table Transcription induction factors for the up-regulated genes. (PDF)

S3 Table Spearman's rank correlation of transcriptome and secertome per node for day 3 and 7. (PDF)

S4 Table Nodes containing genes up-regulated on at least two lignocellulosic substrates and statistically enriched gene annotations. (PDF)

S5 Table Nodes containing genes specifically up-regulated on pine and statistically enriched gene annotations. (PDF)

S1 File Animated transcriptomic topographies. The animation was made based on the transcriptomic topographies made with mean transcription levels per node for each cultivation condition (Fig 2).
(ZIP)

S2 File Enriched CBM1 attached CAZymes in nodes 7 and 14. The output of the hypergenometric test (adjusted p < 0.05).
(CSV)

S3 File Selected six nodes containing the secreted proteins detected. The table contains protein ID, node ID, secretomic observations at day 3 and 7, high transcription, high log2 fold difference, averaged log2 normalized read counts of replicates, log2 transcriptional changes against maltose, CAZyme and other functional annotations.
(XLSX)

S4 File All 456 nodes with detailed information. The large excel spread sheet contains protein ID, node ID, averaged normalized log2 transcript read counts of replicates, log2 transcriptional changes against maltose, specific high transcription, secreted proteins, and corresponding gene annotations (CAZyme, KOG, KEGG, GO).
(XLSX)

S5 File Summary of secretomic information. The excel spread sheet contains protein ID and the number of spectra for all cultivation conditions.
(XLSX)
