# Supplementary material for: The integrative omics of white-rot fungus Pycnoporus coccineus reveals co-regulated CAZymes for orchestrated lignocellulose breakdown
Source: PLoS One. 2017 Apr 10;12(4):e0175528. doi: 10.1371/journal.pone.0175528 (PMC5386290; doi:10.1371/journal.pone.0175528)
Supplement: S1 Fig — (PDF) [file pone.0175528.s001.pdf]

S1 Figure. Growth of P. coccineus CIRM-BRFM310 on the substrates.

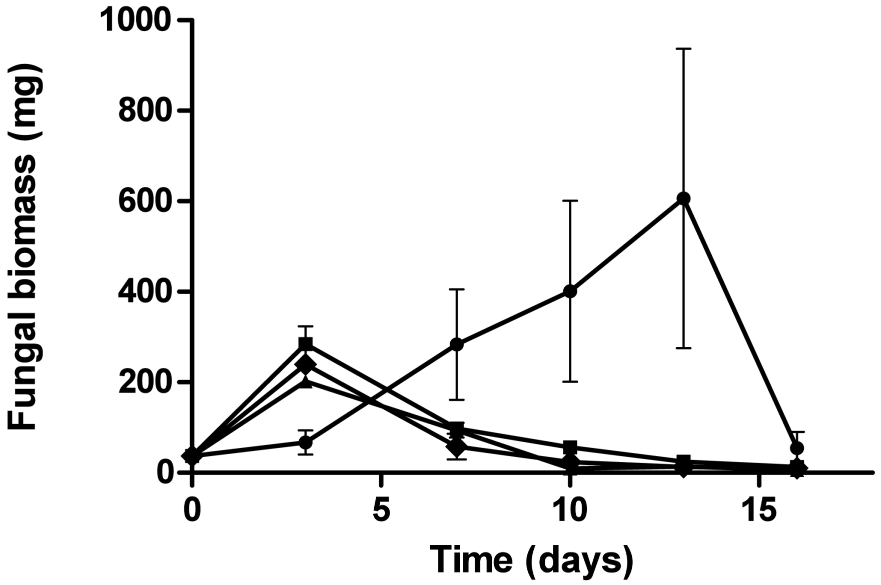

Maltose (circle), wheat straw (square), pine (triangle) and aspen (diamond) determined with the qPCR method (Zhou et al., 2015*).

*Zhou S, Grisel S, Herpoël-Gimbert I, Marie-Noëlle R. A PCR-based method to quantify fungal growth during pretreatment of lignocellulosic biomass. J Microbiol Methods. 2015;115: 67–70. doi:10.1016/j.mimet.2015.05.024
