# Supplementary material for: The integrative omics of white-rot fungus Pycnoporus coccineus reveals co-regulated CAZymes for orchestrated lignocellulose breakdown
Source: PLoS One. 2017 Apr 10;12(4):e0175528. doi: 10.1371/journal.pone.0175528 (PMC5386290; doi:10.1371/journal.pone.0175528)
Supplement: S2 Fig — (PDF) [file pone.0175528.s002.pdf]

S2 Figure. Correlation of normalized read counts from the three biological replicates in four conditions at two time points.

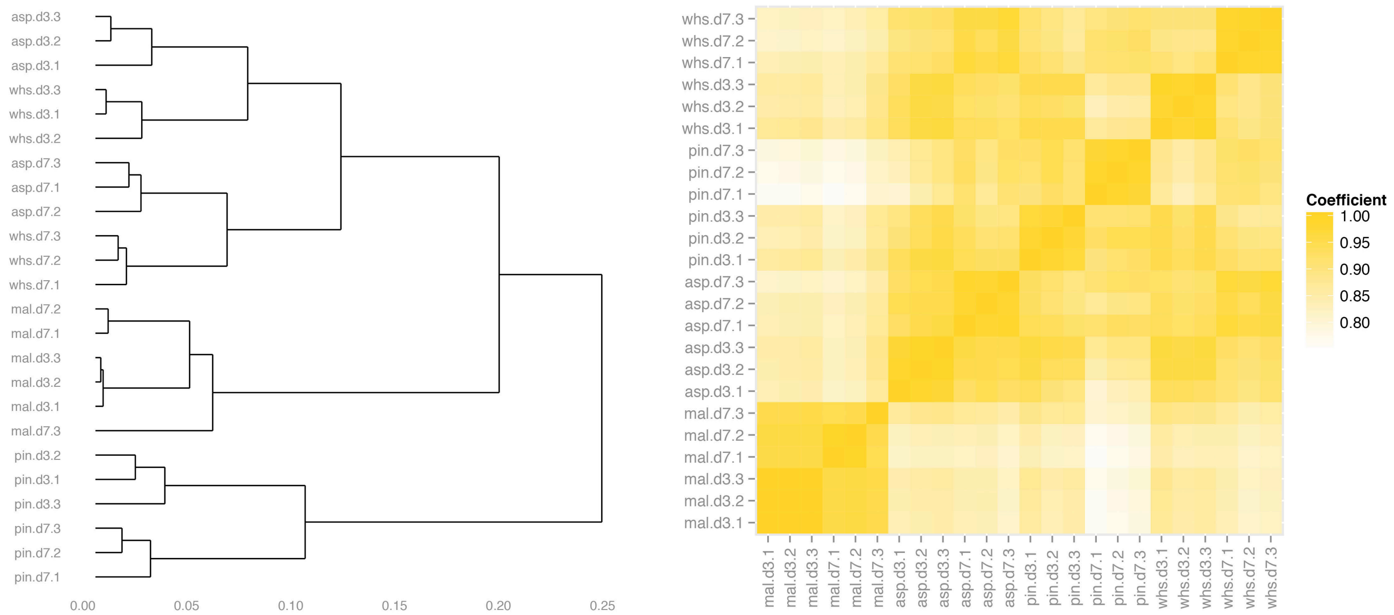

The biological replicates from each condition were highly correlated (> 0.9 Spearman correlation co-efficient). **Left**: Clusters of all biological replicates based on the correlation co-efficient. **Right**: Correlation heatmap of all biological replicates. **mal/asp/pin/whs:** Maltose, Aspen, Pine, Wheat straw. **d3/d7:** Third/seventh day cultures.
