# Supplementary material for: The integrative omics of white-rot fungus Pycnoporus coccineus reveals co-regulated CAZymes for orchestrated lignocellulose breakdown
Source: PLoS One. 2017 Apr 10;12(4):e0175528. doi: 10.1371/journal.pone.0175528 (PMC5386290; doi:10.1371/journal.pone.0175528)
Supplement: S3 Fig — (PDF) [file pone.0175528.s003.pdf]

S3 Figure. Box and density plot of the normalised log2 read counts from three biological replicates in four cultivation conditions at two time points.

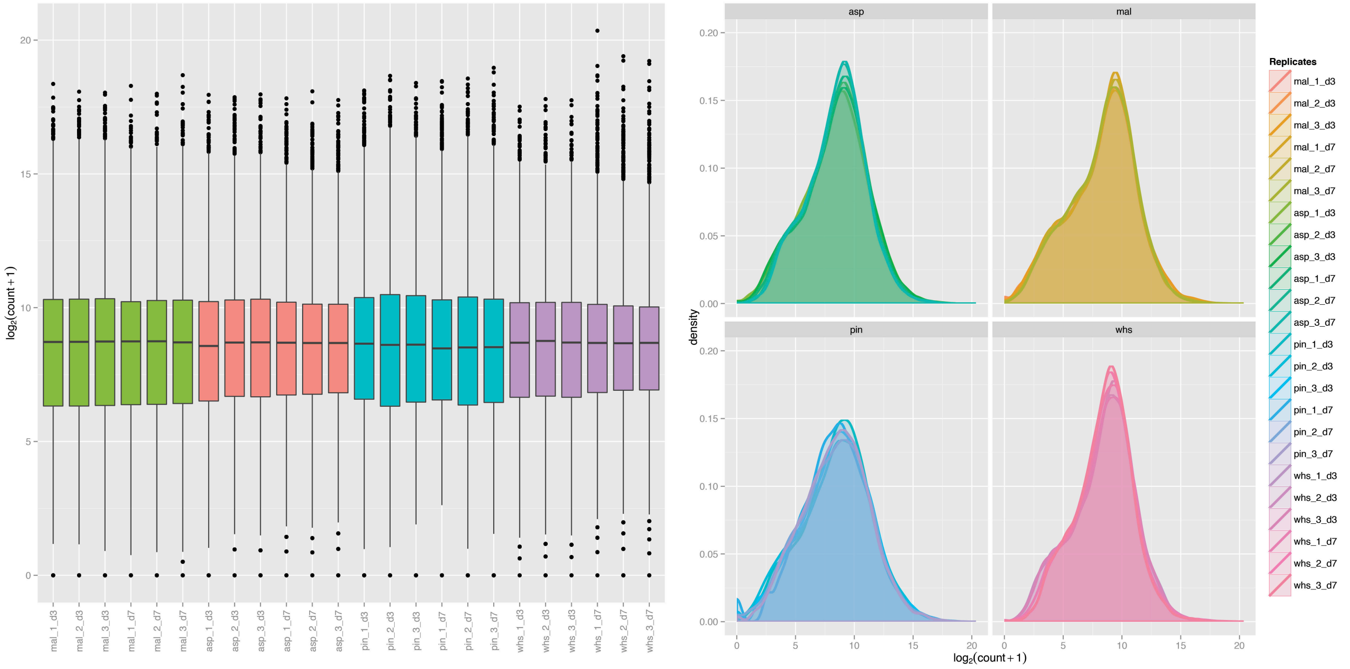

The normalized log2 transformed read count of genes used for the integrated omics models showed similar distributions of log2 read count with the median value of approximately 9 for all 24 samples (left). The shape of the density of the log2 reads was almost identical in each cultivation condition (right). **mal/asp/pin/whs:** Maltose, Aspen, Pine, Wheat straw. **d3/d7:** Third/seventh day cultures.
