# Supplementary material for: The integrative omics of white-rot fungus Pycnoporus coccineus reveals co-regulated CAZymes for orchestrated lignocellulose breakdown
Source: PLoS One. 2017 Apr 10;12(4):e0175528. doi: 10.1371/journal.pone.0175528 (PMC5386290; doi:10.1371/journal.pone.0175528)
Supplement: S4 Fig — (PDF) [file pone.0175528.s004.pdf]

S4 Figure. The normalised log2 transformed read count of housekeeping genes.

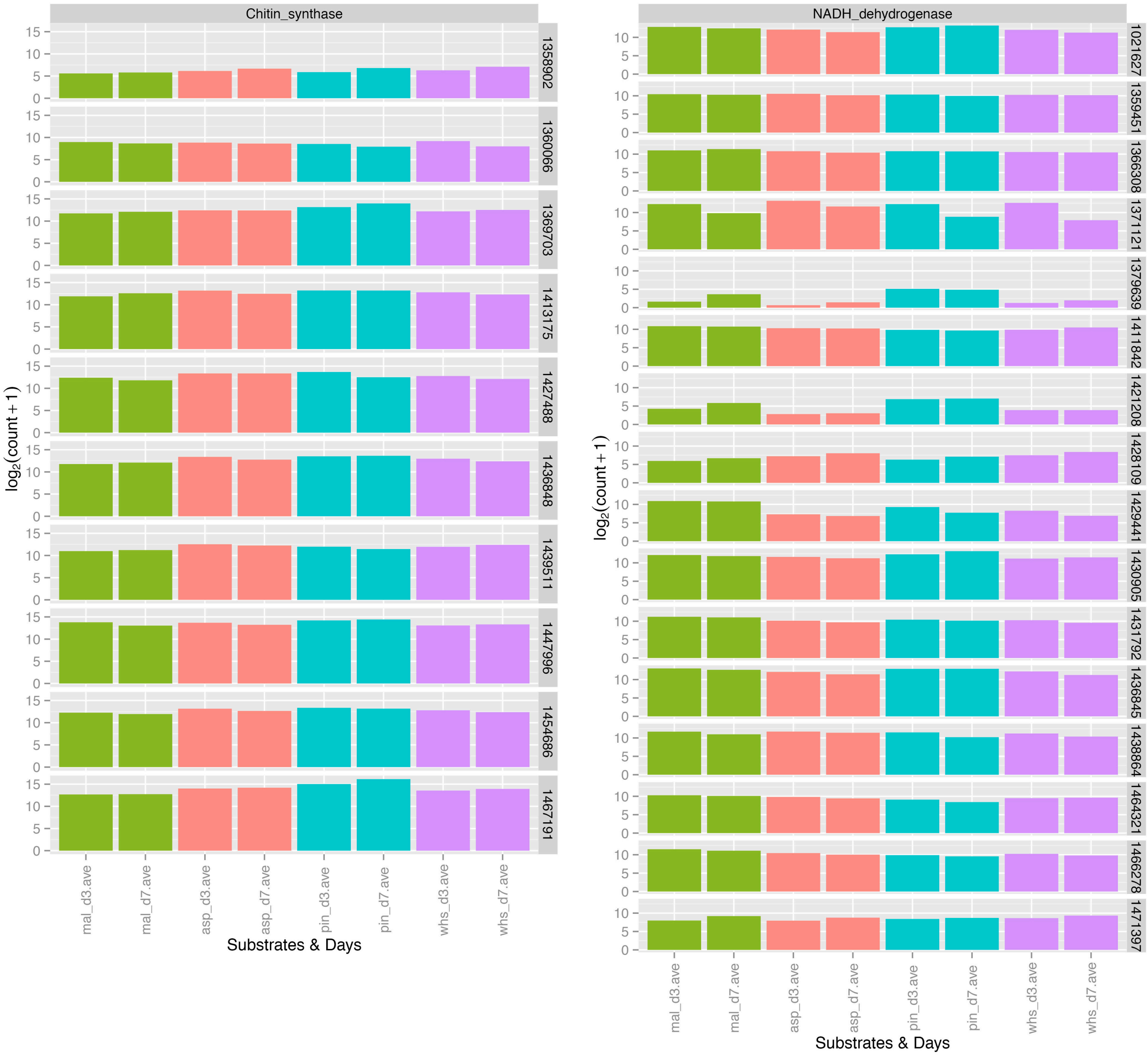

Predicted chitin synthase (10 genes) and NADH dehydrogenase (16 genes) from all conditions - cultivations on four substrates at two time potins. **mal/asp/pin/whs:** Maltose, Aspen, Pine, Wheat straw. **d3/d7:** Third/seventh day cultures.
