# Supplementary material for: The integrative omics of white-rot fungus Pycnoporus coccineus reveals co-regulated CAZymes for orchestrated lignocellulose breakdown
Source: PLoS One. 2017 Apr 10;12(4):e0175528. doi: 10.1371/journal.pone.0175528 (PMC5386290; doi:10.1371/journal.pone.0175528)
Supplement: S5 Fig — (PDF) [file pone.0175528.s005.pdf]

S5 Figure. Transcriptomic profiles of three biological replicates in four cultivation conditions at two time points.

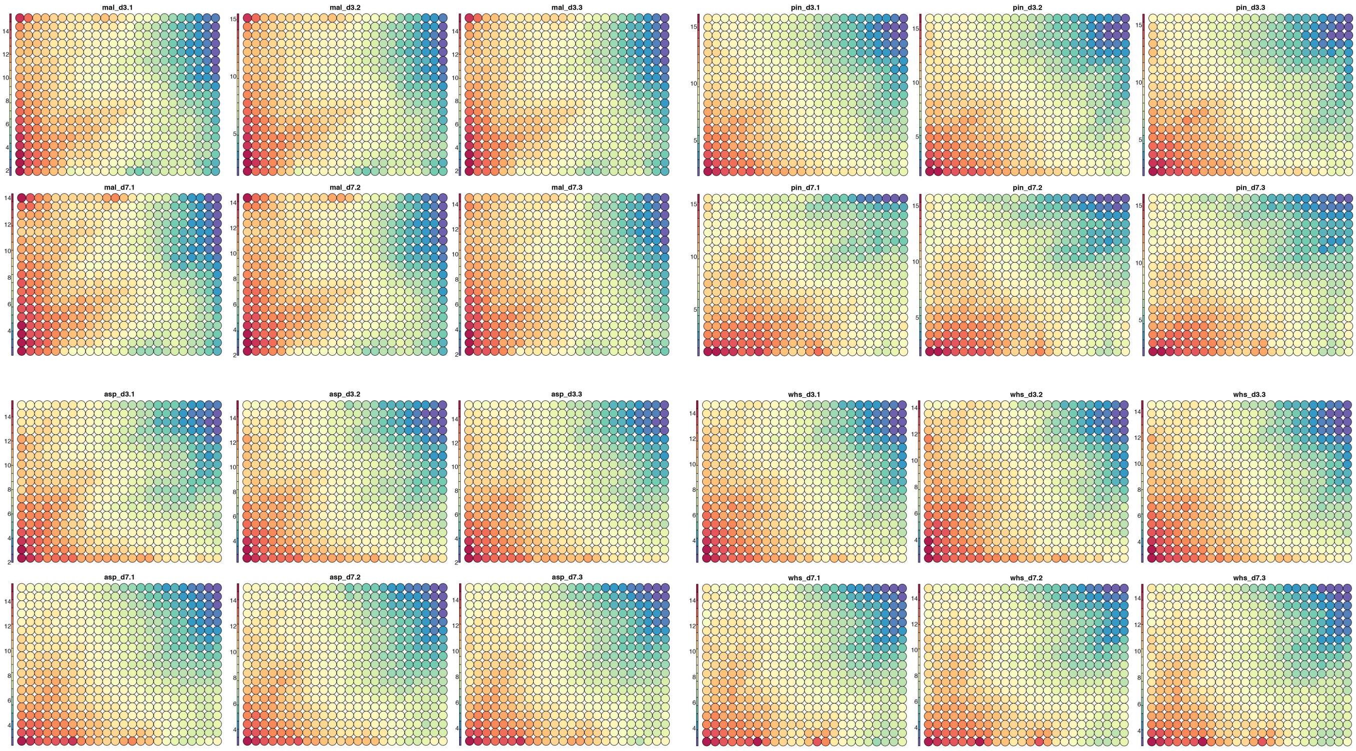

Y axis represents the level of mean log2 read counts per node. **mal/asp/pin/whs:** Maltose, Aspen, Pine, Wheat straw. **d3/d7:** Third/seventh day cultures. The transcriptomic topographies made with individual replicates in each growth condition showed consistent patterns, suggesting that the biological triplicates behaved similarly under each condition. Globally, the highly transcribed nodes were clustered on the left bottom while the lowly transcribed nodes were on the right top corner in the transcriptomic topographies. The transcriptomic responses to maltose showed highly transcribed nodes located on the vertical left edge whereas the responses to the plant substrates shaped rather spread horizontally towards the bottom in the transcriptomic topographies.
