# Supplementary material for: The integrative omics of white-rot fungus Pycnoporus coccineus reveals co-regulated CAZymes for orchestrated lignocellulose breakdown
Source: PLoS One. 2017 Apr 10;12(4):e0175528. doi: 10.1371/journal.pone.0175528 (PMC5386290; doi:10.1371/journal.pone.0175528)
Supplement: S1 Table — (PDF) [file pone.0175528.s006.pdf]

S1 Table. Statistically significant enrichments in gene annotations per node.

NodeID	Specificity	Annotations (GO, KOG, KEGG)
1	Com	sugar:hydrogen symporter activity (GO)<br>beta-1,6-N-acetylglucosaminyltransferase, contains WSC domain; C-type lectin (KOG)<br>L-arabinose isomerase(KEGG)
2	Com	Membrane (GO);<br>Ferric reductase, NADH/NADPH oxidase and related proteins; Chitinase (KOG)
3	Com	1,3-beta-glucan synthase complex; 1,3-beta-glucan synthase activity; saccharopepsin activity; 1,3-beta-glucan biosynthetic process (GO)<br>1,3-beta-glucan synthase/callose synthase catalytic subunit(KOG)<br>1,3-beta-glucan synthase; Saccharopepsin(KEGG)
4	Com	two-component response regulator activity; two-component signal transduction system (phosphorelay); regulation of transcription, DNA-dependent (GO)<br>Sensory transduction histidine kinase; beta-1,6-N-acetylglucosaminyltransferase, contains WSC domain(KOG)
5	Pln	FOG: RRM domain(KOG)
7	Caz, Pln	hydrolase activity, hydrolyzing O-glycosyl compounds; extracellular region; carbohydrate metabolic process; cellulose binding (GO)<br>Glucose dehydrogenase/choline dehydrogenase/mandelonitrile lyase (GMC oxidoreductase family) (KOG)<br>Choline dehydrogenase(KEGG)
8	Caz, Pln	Glucose dehydrogenase/choline dehydrogenase/mandelonitrile lyase (GMC oxidoreductase family) (KOG)<br>Choline dehydrogenase(KEGG)
9	Pln, Det	Cytochrome P450 CYP4/CYP19/CYP26 subfamilies(KOG)
12	Pln	Aldo/keto reductase family proteins(KOG)<br>With NAD(+) or NADP(+) as acceptor(KEGG)
14	Caz, Pln	polysaccharide catabolic process; hydrolase activity, hydrolyzing O-glycosyl compounds; extracellular region; carbohydrate metabolic process; cellulase activity; cellulose binding (GO)<br>von Willebrand factor and related coagulation proteins(KOG)
15	Caz, Pln	hydrolase activity, hydrolyzing O-glycosyl compounds; polygalacturonase activity; carbohydrate metabolic process; cellulose binding; galacturan 1,4-alpha-galacturonidase activity (GO)<br>Galacturan 1,4-alpha-galacturonidase(KEGG)
16	Pln	cellulose binding (GO)
19	Pln	Oxidoreductases(KEGG)
21		3-keto sterol reductase activity; aldo-keto reductase activity; mevaldate reductase activity; gluconate dehydrogenase activity; steroid dehydrogenase activity; epoxide dehydrogenase activity; 5-exo-hydroxycamphor dehydrogenase activity; 2-hydroxytetrahydrofuran dehydrogenase activity; phenylcoumaran benzylic ether reductase activity; steroid dehydrogenase activity, acting on the CH-OH group of donors, NAD or NADP as acceptor; steroid dehydrogenase activity, acting on the CH-CH group of donors; (R)-2-hydroxyisocaproate dehydrogenase activity; 3-ketoglucose-reductase activity; (R)-2-hydroxyglutarate dehydrogenase activity (GO)<br>With NAD(+) or NADP(+) as acceptor(KEGG)
22	Pln	peroxidase activity (GO)
23	Pln	oxidoreductase activity (GO)<br>Permease of the major facilitator superfamily(KOG)
24	Pln	protein-S-isoprenylcysteine O-methyltransferase activity; G-protein coupled receptor activity; C-terminal protein amino acid methylation (GO)
25	Com	Aspartyl protease(KOG)<br>H(+)-transporting two-sector ATPase(KEGG)
28	Com	motor activity; chitin synthase activity; myosin complex (GO)<br>Chitin synthase/hyaluronan synthase (glycosyltransferases) (KOG)<br>Chitin synthase(KEGG)
33	Pln	cAMP-dependent protein kinase complex (GO)
39	Pln	L-arabinose isomerase activity (GO)<br>Predicted transporter (major facilitator superfamily) (KOG)<br>L-arabinose isomerase(KEGG)
40	Pln, Det	Cytochrome P450 CYP2 subfamily; Predicted transporter (major facilitator superfamily) (KOG)<br>L-arabinose isomerase(KEGG)
44	Det	Cytochrome P450 CYP2 subfamily(KOG)
47	Pln, Det	salicylate 1-monooxygenase activity (GO)<br>Kynurenine 3-monooxygenase and related flavoprotein monooxygenases(KOG)<br>Cholesterol 7-alpha-monooxygenase | Salicylate 1-monooxygenase(KEGG)
49	Com	pentose-phosphate shunt; transition metal ion binding(GO)
53	Com	septin complex(GO)
55	Pln	Phosphotransferases with an alcohol group as acceptor(KEGG)
56	Pln	MAP kinase kinase kinase activity(GO)
57	Pln	Splicing coactivator SRm160/300, subunit SRm300(KOG)<br>Glucan 1,4-alpha-glucosidase(KEGG)
58	Pln	p21-activated serine/threonine protein kinase; Splicing coactivator SRm160/300, subunit SRm300(KOG)<br>Glucan 1,4-alpha-glucosidase(KEGG)
59		SWI-SNF chromatin-remodeling complex protein(KOG)
60		von Willebrand factor and related coagulation proteins(KOG)
63	Det	unspecific monooxygenase activity(GO)<br>Cytochrome P450 CYP2 subfamily(KOG)<br>Unspecific monooxygenase(KEGG)
70		FMN binding(GO)
73	Com	structural constituent of ribosome; intracellular; ribosome; translation(GO)<br>Tripeptidyl-peptidase I(KEGG)
74	Com	aspartate transaminase activity; cellular carbohydrate metabolic process(GO)<br>4-aminobutyrate aminotransferase | Aspartate aminotransferase | Ornithine--oxo-acid aminotransferase | Acetylornithine aminotransferase | Glutamine-fructose-6-phosphate transaminase (isomerizing) (KEGG)

75	Com	Glucose dehydrogenase/choline dehydrogenase/mandelonitrile lyase (GMC oxidoreductase family) (KOG)<br>Choline dehydrogenase(KEGG)
76	Com	oxidoreductase activity, acting on NADH or NADPH(GO)<br>Ras-related small GTPase, Rho type(KOG)
77	Com	Tripeptidyl-peptidase I(KEGG)
78	Pln	Vigilin(KOG)
80	Pln	Unspecific monooxygenase(KEGG)
83		glucan 1,4-alpha-glucosidase activity(GO)<br>Uncharacterized conserved protein(KOG)<br>Glucan 1,4-alpha-glucosidase(KEGG)
84		UDP-N-acetylmuramoylalanyl-D-glutamyl-2,6-diaminopimelate-D-alanyl-D-alanine ligase activity; ribosomal S6-glutamic acid ligase activity; coenzyme F420-0 gamma-glutamyl ligase activity; coenzyme F420-2 alpha-glutamyl ligase activity(GO)<br>Splicing coactivator SRm160/300, subunit SRm300(KOG)<br>Acid--D-amino-acid ligases (peptide synthases) (KEGG)
85		Splicing coactivator SRm160/300, subunit SRm300(KOG)
90	Det	Multidrug resistance-associated protein/mitoxantrone resistance protein, ABC superfamily(KOG)
97	Com	structural constituent of ribosome; intracellular; ribosome; translation; small ribosomal subunit(GO)<br>Acting on paired donors, with incorporation or reduction of(KEGG)
102	Pln	dolichyl-diphosphooligosaccharide-protein glycotransferase activity(GO)<br>Dolichyl-diphosphooligosaccharide--protein glycosyltransferase(KEGG)
107		Exo-alpha-sialidase(KEGG)
109		Exo-alpha-sialidase(KEGG)
110		Serine/threonine protein kinase; Adaptor protein Enigma and related PDZ-LIM proteins(KOG)
111		Splicing coactivator SRm160/300, subunit SRm300(KOG)
117	Det	Cytochrome P450 CYP2 subfamily(KOG)
121	Com	structural constituent of ribosome; intracellular; ribosome; translation(GO)
123	Com	nucleosome; nucleosome assembly(GO)<br>Histone H4(KOG)
125	Pln	Serine/threonine specific protein phosphatase(KEGG)
126	Pln	Long-chain acyl-CoA synthetases (AMP-forming) (KOG)<br>Long-chain-fatty-acid--CoA ligase(KEGG)
128		ribonuclease III activity(GO)
129		salicylate 1-monooxygenase activity(GO)<br>Cholesterol 7-alpha-monooxygenase | Salicylate 1-monooxygenase(KEGG)
131		Serine/threonine specific protein phosphatase(KEGG)
132		Serine/threonine protein kinase(KOG)
133		glucan 1,4-alpha-glucosidase activity(GO)<br>Glucan 1,4-alpha-glucosidase(KEGG)
135		Kelch repeat-containing proteins(KOG)
137		Predicted histone tail methylase containing SET domain(KOG)
138		RNA polymerase II, large subunit(KOG)
141	Det	monooxygenase activity; iron ion binding; electron transport; heme binding(GO)<br>Cytochrome P450 CYP2 subfamily(KOG)<br>Unspecific monooxygenase(KEGG)
145	Com	structural constituent of ribosome; intracellular; ribosome; translation(GO)
146	Com	hydrogen-transporting two-sector ATPase activity; cytochrome-c oxidase activity; aa3-type cytochrome c oxidase; ba3-type cytochrome c oxidase; caa3-type cytochrome c oxidase; cbb3-type cytochrome c oxidase; ATP synthesis coupled proton transport; proton-transporting two-sector ATPase complex; proton-transporting ATP synthase complex; hydrogen ion transporting ATP synthase activity, rotational mechanism; hydrogen ion transporting ATPase activity, rotational mechanism(GO)<br>Cytochrome-c oxidase; H(+)-transporting two-sector ATPase(KEGG)
151		sulfite reductase (NADPH) activity(GO)<br>Tripeptidyl-peptidase I(KEGG)
152		protein kinase CK2 complex; protein kinase CK2 regulator activity(GO)<br>Casein kinase II, beta subunit(KOG)
159		Splicing coactivator SRm160/300, subunit SRm160 (contains PWI domain) (KOG)<br>Acid--D-amino-acid ligases (peptide synthases) (KEGG)
161		glucan 1,4-alpha-glucosidase activity(GO)<br>von Willebrand factor and related coagulation proteins(KOG)<br>Glucan 1,4-alpha-glucosidase(KEGG)
165		Serine/threonine protein kinase(KOG)
166		lipid metabolic process; oxidoreductase activity, acting on paired donors, with oxidation of a pair of donors resulting in the reduction of molecular oxygen to two molecules of water; phosphatidylcholine desaturase activity(GO)<br>Phosphatidylcholine desaturase(KEGG)
168	Pln	Notchless-like WD40 repeat-containing protein(KOG)
169	Mal	structural constituent of ribosome; intracellular; ribosome; translation(GO)<br>NADH-dehydrogenase (ubiquinone) (KOG)
171		ligase activity, forming aminoacyl-tRNA and related compounds; tRNA aminoacylation(GO)
177		phosphatidate cytidylyltransferase activity(GO)<br>Cholesterol transport protein (Niemann-Pick C disease protein); FOG: PPR repeat(KOG)

186		Serine/threonine protein kinase(KOG)
193	Mal	glycolysis(GO)
194	Mal	ATPase activity, coupled to transmembrane movement of substances(GO)<br>Adenosinetriphosphatase(KEGG)
195		transcription factor binding(GO)
196		Iron/ascorbate family oxidoreductases(KOG)
199		Nucleolar GTPase/ATPase p130(KOG)
201		protein import into nucleus, docking; nuclear pore; intracellular protein transport; protein transporter activity(GO)
202		Ubiquitin thiolesterase(KEGG)
218		endopeptidase activity; threonine endopeptidase activity; proteasome endopeptidase activity(GO)<br>Proteasome endopeptidase complex(KEGG)
222		WASP-interacting protein VRP1/WIP, contains WH2 domain(KOG)
225	Det	Cytochrome P450 CYP4/CYP19/CYP26 subfamilies(KOG)
226		Calcium-responsive transcription coactivator(KOG)
227		Anaphase promoting complex, Cdc20, Cdh1, and Ama1 subunits(KOG)
229		Adenosinetriphosphatase(KEGG)
242		endopeptidase activity; proteasome core complex<br>Proteasome endopeptidase complex(KEGG)
243		ARF GTPase activator activity; regulation of ARF GTPase activity(GO)
244		Gluconate transport-inducing protein(KOG)<br>2-acetyl-1-alkylglycerophosphocholine esterase(KEGG)
252		Methyltransferases(KEGG)
260		regulation of nitrogen utilization; transcription repressor activity(GO)
261		ribonuclease H activity(GO)<br>Serine/threonine protein kinase; Ribonuclease H(KOG)
265		threonine endopeptidase activity; proteasome endopeptidase activity; cytoplasm; glutamine metabolic process(GO)<br>Acting on peptide bonds (peptide hydrolases); Proteasome endopeptidase complex (KEGG)
267		NADH dehydrogenase (ubiquinone) activity(GO)
270		RNA processing(GO)<br>Reductases with broad range of substrate specificities(KOG)
271		Acid--D-amino-acid ligases (peptide synthases) (KEGG)
272		Iron/ascorbate family oxidoreductases(KOG)
274		sugar binding(GO)
277		Adenosinetriphosphatase(KEGG)
278		DNA-directed DNA polymerase(KEGG)
285		Nucleolar GTPase/ATPase p130(KOG)
289		ubiquitin-protein ligase activity; regulation of ubiquitin-protein ligase activity; regulation of ubiquitin-protein ligase activity during meiotic cell cycle; positive regulation of ubiquitin-protein ligase activity; negative regulation of ubiquitin-protein ligase activity(GO)<br>Ubiquitin--protein ligase(KEGG)
290		Ubiquitin--protein ligase(KEGG)
291		Rab GTPase activator activity; regulation of Rab GTPase activity(GO)
292		ligase activity(GO)
300		Helicase-like transcription factor HLTF/DNA helicase RAD5, DEAD-box superfamily(KOG)
313		nucleosome; nucleosome assembly(GO)<br>Histones H3 and H4(KOG)
318		Transcription regulator XNP/ATRX, DEAD-box superfamily(KOG)
320		cis-Golgi network(GO)<br>Histone acetyltransferase(KEGG)
324		Myosin class II heavy chain(KOG)
325		telomerase activity; RNA helicase activity; DNA-directed DNA polymerase activity; DNA primase activity; sulfate adenylyltransferase activity; RNA guanylyltransferase activity; tRNA guanylyltransferase activity; mannose-phosphate guanylyltransferase activity; DNA/RNA helicase activity; CTP:2,3-di-O-geranylgeranyl-sn-glycero-1-phosphate cytidyltransferase; phospholactate guanylyltransferase activity(GO)<br>Nucleotidyltransferases(KEGG)
327		base-excision repair(GO)<br>In phosphorous-containing anhydrides(KEGG)
330		Transcriptional regulator(KOG)
332		Exo-alpha-sialidase(KEGG)
335		cathepsin D activity(GO)<br>HMG-box transcription factor; Aspartyl protease(KOG)<br>Cathepsin D(KEGG)

337		Histone 2A(KOG)
347		DNA-directed DNA polymerase(KEGG)
355		beta-1,6-N-acetylglucosaminyltransferase, contains WSC domain(KOG)
356		Collagens (type IV and type XIII), and related proteins(KOG)
359		nuclease activity; endonuclease activity(GO)
367		Peptidylprolyl isomerase(KEGG)
368		Synaptic vesicle transporter SVOP and related transporters (major facilitator superfamily) (KOG)
371		DNA ligase (ATP) activity(GO)<br>DNA ligase (ATP) (KEGG)
374		DNA-directed DNA polymerase(KEGG)
379		protein-tyrosine kinase activity; protein amino acid phosphorylation(GO)
380		beta-1,6-N-acetylglucosaminyltransferase, contains WSC domain(KOG)
386	Det	Cytochrome P450 CYP4/CYP19/CYP26 subfamilies(KOG)
392		ER to Golgi vesicle-mediated transport(GO)
393		DNA ligase (ATP) (KEGG)
394		aspartate-tRNA ligase activity(GO)<br>Aspartate--tRNA ligase(KEGG)
396		Predicted histone tail methylase containing SET domain(KOG)<br>Exoribonucleases producing 5'-phosphomonoesters(KEGG)
398		ribonuclease P activity; ribonuclease activity(GO)<br>Ribonuclease P(KEGG)
400		protein-tyrosine kinase activity(GO)
405		alcohol dehydrogenase activity; alcohol dehydrogenase activity, metal ion-independent; alcohol dehydrogenase activity, zinc-dependent; alcohol dehydrogenase activity, iron-dependent(GO)<br>3-oxoacyl-[acyl-carrier protein] reductase | Acylglycerone-phosphate reductase | Carbonyl reductase (NADPH) | Alcohol dehydrogenase | Cinnamyl-alcohol dehydrogenase | L-iditol 2-dehydrogenase | Myo-inositol 2-dehydrogenase | L-threonine 3-dehydrogenase | 2-dehydropantoate 2-reductase | 3-beta-hydroxy-4-alpha-methylcholestenecarboxylate 3-dehydrogenase (decarboxylating) | 2-deoxy-D-gluconate 3-dehydrogenase | 3-dehydrosphinganine reductase | 3-hydroxybutyryl-CoA dehydrogenase | D-xylose 1-dehydrogenase (NADP+) | 11-beta-hydroxysteroid dehydrogenase(KEGG)
406		Glutathione S-transferase(KOG)
408		FOG: Zn-finger(KOG)
414		DNA-directed RNA polymerase(KEGG)
433	Mal	anthranilate synthase activity; tryptophan metabolic process; metabolic process; aromatic amino acid family biosynthetic process(GO)<br>Zinc-binding oxidoreductase; Voltage-gated shaker-like K+ channel, subunit beta/KCNAB(KOG)<br>Pyridoxine 4-dehydrogenase(KEGG)
434	Mal	oxidoreductase activity; pyridoxine 4-dehydrogenase activity(GO)<br>Voltage-gated shaker-like K+ channel, subunit beta/KCNAB(KOG)<br>Pyridoxine 4-dehydrogenase(KEGG)
440		DNA-directed RNA polymerase(KEGG)
444		Glutathione S-transferase; Flavonol reductase/cinnamoyl-CoA reductase(KOG)
448		Oxidoreductases (KEGG)
449		Ca2+-modulated nonselective cation channel polycystin(KOG)
450		Predicted short chain-type dehydrogenase(KOG)

The gene annotation datasets were based on the Gene Ontology (GO), Kyoto Encyclopedia of Genes and Genomes (KEGG), and EuKaryotic Orthologous Groups (KOG; Ogata et al., 1999; Tatusov et al., 2003; Blake et al., 2015). **Com**: Nodes commonly highly transcribed among all cultivation conditions regardless of the time points. **Mal**: Nodes highly transcribed on maltose regardless of the time points. **Pln**: Nodes specifically transcribed on plant substrates; aspen, pine, wheat straw regardless of the time points. **Caz**: Nodes with enriched Cazymes. **Det**: Nodes with enriched genes for detoxification processes.
