# Supplementary material for: The integrative omics of white-rot fungus Pycnoporus coccineus reveals co-regulated CAZymes for orchestrated lignocellulose breakdown
Source: PLoS One. 2017 Apr 10;12(4):e0175528. doi: 10.1371/journal.pone.0175528 (PMC5386290; doi:10.1371/journal.pone.0175528)
Supplement: S2 Table — (PDF) [file pone.0175528.s007.pdf]

S2 Table. Transcription induction factors for the up-regulated genes.

Protein ID	CAZyme	Node	Transcription induction factor					
			Day 3			Day 7		
			Asp vs Mal	Pin vs Mal	Whs vs Mal	Asp vs Mal	Pin vs Mal	Whs vs Mal
Auxiliary Activity enzymes								
1468611	AA2 (MnP)	7	476	336	206	129	43	27
1431101	AA2 (LiP)	15	652	9	12	277	2	18
1465271	AA3_3	7	37	23	10	37	39	29
1463000	AA3_2	8	5	6	4	32	46	20
1465734	AA3_2	8	8	14	6	16	55	26
1480943	AA5_1 (Glx)	8	28	8	10	43	24	5
1401955	AA8-AA3_1	7	84	42	67	64	84	351
1374028	AA9	7	66	50	115	82	47	290
1382161	AA9-CBM1	7	42	22	15	89	60	401
1428145	AA9-CBM1	7	35	15	20	31	47	152
793241	AA9	15	11	17	13	31	20	198
1369390	AA9	15	30	24	8	13	40	96
1417214	AA9	15	82	26	104	115	22	412
1430659	AA9	15	56	14	5	133	78	233
1466495	AA9	15	11	54	109	37	45	112
Carbohydrate Esterases								
1377173	CBM1-CE1	14	30	47	10	56	199	241
1392142	CBM1-CE1	14	16	3	2	21	15	78
1433098	CE4	8	3	18	5	9	23	15
1433098	CE4	8	3	18	5	9	23	15
1438246	CE8	14	19	27	29	17	24	26
1470260	CBM1-CE15	14	16	9	8	70	82	312
1439328	CBM1-CE16	7	27	51	58	46	139	208
1429512	CE16	14	49	19	6	24	44	35
1437703	CE16	15	79	15	9	34	45	30
Carbohydrate Hydrolases								
1370248	GH1	7	15	10	25	53	26	133
1468141	GH3	8	12	14	15	10	24	21
1411666	GH3	14	33	19	29	23	18	53
1375024	CBM1-GH5_5	14	41	46	24	44	85	267
1429791	CBM1-GH5_5	15	63	33	19	25	71	145
1359888	CBM1-GH5_7	7	40	50	22	34	145	227
1357326	CBM1-GH6	7	89	58	19	48	113	221
1424818	GH7	7	46	60	24	53	36	230
1366028	GH7	15	50	8	15	72	15	48
1389216	GH7	15	67	36	39	9	25	34
1435885	CBM1-GH10	7	20	19	7	115	100	328
1437837	CBM1-GH10	14	36	30	14	24	96	206
1358049	GH12	14	37	83	40	25	75	176
1375723	GH12	14	28	23	4	27	15	322
1445188	GH16	7	6	12	9	54	62	54
1370654	GH28	8	25	35	31	22	49	76
1377553	GH28	14	21	19	9	13	17	33
1439310	GH28	15	37	45	54	31	58	63
1446047	GH28	15	18	10	18	23	10	17
1357645	GH30	7	11	19	6	24	254	22
408613	GH43	14	12	26	3	13	39	54
1435501	GH43	14	47	111	39	12	102	58
1433077	GH45	14	156	129	90	72	128	332
1357629	GH51	7	24	25	25	20	38	13
1363671	GH53	14	101	34	24	65	83	128
1434191	GH78	14	12	26	11	17	102	37
1426831	GH115	14	141	47	45	44	58	91
1467772	GH131-CBM1	14	56	35	29	57	58	307
Carbohydrate Binding Modules								
1472584	CBM1	15	48	13	6	30	31	212
Polysaccharide Lyases								
1444442	PL8_4	7	14	22	5	17	28	42

MnP: Manganese Peroxidases, **LiP**: Lignin Peroxidase, **Glx**: Glyoxal Oxidase. **Blue highlight**: Auxiliary Activity enzymes coding genes with a decrease in induction factor at Day 7 in comparison to Day 3. **Mal/Asp/Pin/Whs:** Maltose, Aspen, Pine, Wheat straw.
