# Supplementary material for: The integrative omics of white-rot fungus Pycnoporus coccineus reveals co-regulated CAZymes for orchestrated lignocellulose breakdown
Source: PLoS One. 2017 Apr 10;12(4):e0175528. doi: 10.1371/journal.pone.0175528 (PMC5386290; doi:10.1371/journal.pone.0175528)
Supplement: S3 Table — (PDF) [file pone.0175528.s008.pdf]

S3 Table. **Spearman's rank correlation of transcriptome and secertome per node for day 3 and 7**.

	Day 3 transcriptome	Day 7 transcriptome
Maltose Day3 secretome	0.30 (0.27)	0.31 (0.27)
Maltose Day7 secretome	0.30 (0.34)	0.30 (0.34)
Aspen Day3 secretome	0.44 (0.50)	0.42 (0.51)
Aspen Day7 secretome	0.42 (0.41)	0.43 (0.43)
Pine Day3 secretome	0.36 (0.35)	0.35 (0.38)
Pine Da7 secretome	0.44 (0.44)	0.43 (0.44)
Wheat straw Day3 secretome	0.43 (0.49)	0.43 (0.52)
Wheat straw Day7 secretome	0.41 (0.36)	0.43 (0.45)

Left values: Genome-wide transcriptome and corresponding secretome. **Right values in parentheses**: Selected genes for secreted proteins only. The mean transcription level of all nodes was used as a transcriptomic representation and the frequency of proteins secreted was used as a secretomic representation. $p < 0.001$.
