# Supplementary material for: The integrative omics of white-rot fungus Pycnoporus coccineus reveals co-regulated CAZymes for orchestrated lignocellulose breakdown
Source: PLoS One. 2017 Apr 10;12(4):e0175528. doi: 10.1371/journal.pone.0175528 (PMC5386290; doi:10.1371/journal.pone.0175528)
Supplement: S4 Table — (PDF) [file pone.0175528.s009.pdf]

S4 Table. Nodes containing genes up-regulated on at least two lignocellulosic substrates and statistically enriched gene annotations.

Node	Day3	Day7	Database	Enriched Terms
5	APW	APW	KOG	FOG RRM domain
7	APW	APW	GO	hydrolase activity, hydrolyzing O-glycosyl compounds; extracellular region; carbohydrate metabolic process; cellulose binding
7	APW	APW	KOG	Glucose dehydrogenase/choline dehydrogenase/mandelonitrile lyase (GMC oxidoreductase family)
7	APW	APW	KEGG	Choline dehydrogenase
8	APW	APW	KOG	Glucose dehydrogenase/choline dehydrogenase/mandelonitrile lyase (GMC oxidoreductase family)
8	APW	APW	KEGG	Choline dehydrogenase
9	APW	APW	KOG	Cytochrome P450 CYP4/CYP19/CYP26 subfamilies
12	APW	A	KOG	Aldo/keto reductase family proteins
12	APW	A	KEGG	With NAD(+) or NADP(+) as acceptor
14	APW	APW	GO	polysaccharide catabolic process; hydrolase activity, hydrolyzing O-glycosyl compounds; extracellular region; carbohydrate metabolic process; cellulase activity; cellulose binding
15	APW	APW	GO	hydrolase activity, hydrolyzing O-glycosyl compounds; polygalacturonase activity; carbohydrate metabolic process; cellulose binding; galacturan 1,4-alpha-galacturonidase activity
15	APW	APW	KOG	von Willebrand factor and related coagulation proteins
15	APW	APW	KEGG	Galacturan 1,4-alpha-galacturonidase
16	APW	APW	GO	cellulose binding
19	APW		KEGG	Oxidoreductases
22	AW		GO	peroxidase activity
23	APW	A	GO	oxidoreductase activity
23	APW	A	KOG	Permease of the major facilitator superfamily
24	APW	APW	GO	protein-S-isoprenylcysteine O-methyltransferase activity; G-protein coupled receptor activity; C-terminal protein amino acid methylation
39	AP	APW	GO	L-arabinose isomerase activity
39	AP	APW	KOG	Predicted transporter (major facilitator superfamily)
39	AP	APW	KEGG	L-arabinose isomerase
40	APW	APW	KOG	Cytochrome P450 CYP2 subfamily; Predicted transporter (major facilitator superfamily)
40	APW	APW	KEGG	L-arabinose isomerase
47	APW		GO	salicylate 1-monooxygenase activity
47	APW		KOG	Kynurenine 3-monooxygenase and related flavoprotein monooxygenases
47	APW			Cholesterol 7-alpha-monooxygenase | Salicylate 1-monooxygenase
55	AP	AP	KEGG	Phosphotransferases with an alcohol group as acceptor
63		PW	GO	unspecific monooxygenase activity
63		PW	KOG	Cytochrome P450 CYP2 subfamily
63		PW	KEGG	Unspecific monooxygenase
78	P	PW	KOG	Vigilin
125	AP	P	KEGG	Serine/threonine specific protein phosphatase
168	APW	APW	KOG	Notchless-like WD40 repeat-containing protein

A: Aspen. **P**: Pine. **W**: Wheat straw. **GO**: The Gene Ontology. **KEGG**: Kyoto Encyclopedia of Genes and Genomes. **KOG**: EuKaryotic Orthologous Groups.
