# Supplementary material for: The integrative omics of white-rot fungus Pycnoporus coccineus reveals co-regulated CAZymes for orchestrated lignocellulose breakdown
Source: PLoS One. 2017 Apr 10;12(4):e0175528. doi: 10.1371/journal.pone.0175528 (PMC5386290; doi:10.1371/journal.pone.0175528)
Supplement: S5 Table — (PDF) [file pone.0175528.s010.pdf]

S5 Table. Nodes containing genes specifically up-regulated on pine and statistically enriched gene annotations.

Node	Day3	Day7	Database	Enriched Terms
2		P	GO	Membrane
			KOG	Ferric reductase, NADH/NADPH oxidase and related proteins; Chitinase
3		P	GO	1,3-beta-glucan synthase complex; 1,3-beta-glucan synthase activity; saccharopepsin activity; 1,3-beta-glucan biosynthetic process
			KOG	1,3-beta-glucan synthase/callose synthase catalytic subunit
			KEGG	1,3-beta-glucan synthase; Saccharopepsin
4		P	GO	two-component response regulator activity; two-component signal transduction system (phosphorelay); regulation of transcription, DNA-dependent
			KOG	Sensory transduction histidine kinase; beta-1,6-N-acetylglucosaminyltransferase, contains WSC domain
33	P	P	GO	cAMP-dependent protein kinase complex
56	P	P	GO	MAP kinase kinase kinase activity
57	P	P	KOG	Splicing coactivator SRm160/300, subunit SRm300
			KEGG	Glucan 1,4-alpha-glucosidase
58		P	KOG	p21-activated serine/threonine protein kinase; Splicing coactivator SRm160/300, subunit SRm300
			KEGG	Glucan 1,4-alpha-glucosidase
80		P	KEGG	Unspecific monooxygenase
102		P	GO	dolichyl-diphosphooligosaccharide-protein glycotransferase activity
			KEGG	Dolichyl-diphosphooligosaccharide--protein glycosyltransferase
126	P		KOG	Long-chain acyl-CoA synthetases (AMP-forming)
			KEGG	Long-chain-fatty-acid--CoA ligase

P: Pine. **GO**: The Gene Ontology. **KEGG**: Kyoto Encyclopedia of Genes and Genomes. **KOG**: EuKaryotic Orthologous Groups.
