# Supplementary figures and images for: The integrative omics of white-rot fungus Pycnoporus coccineus reveals co-regulated CAZymes for orchestrated lignocellulose breakdown
Source: PLoS One. 2017 Apr 10;12(4):e0175528. doi: 10.1371/journal.pone.0175528 (PMC5386290; doi:10.1371/journal.pone.0175528)

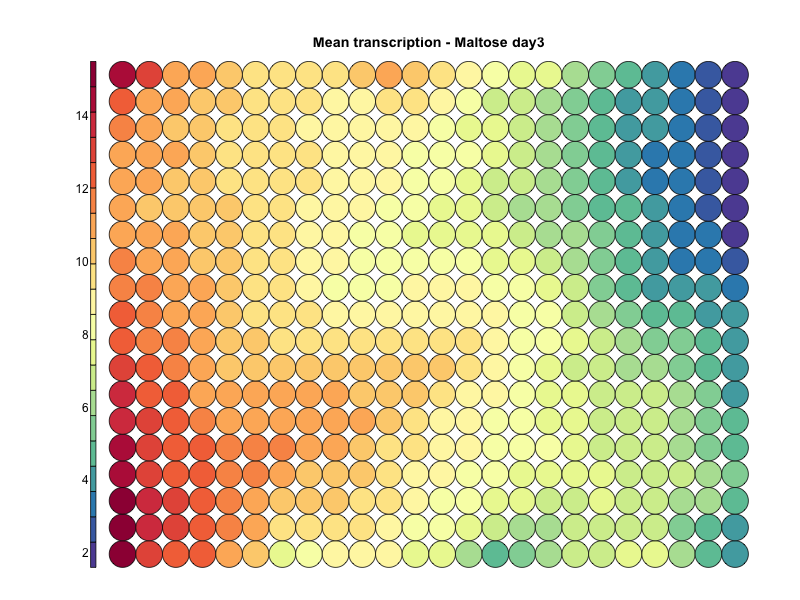

Supplement: S1 File — The animation was made based on the transcriptomic topographies made with mean transcription levels per node for each cultivation condition (Fig 2). (ZIP) [file pone.0175528.s011.zip › S1 File/Pycco310_MeanTranscript_dir/Pycco310_Step10.4_MeanTranscript1.png]

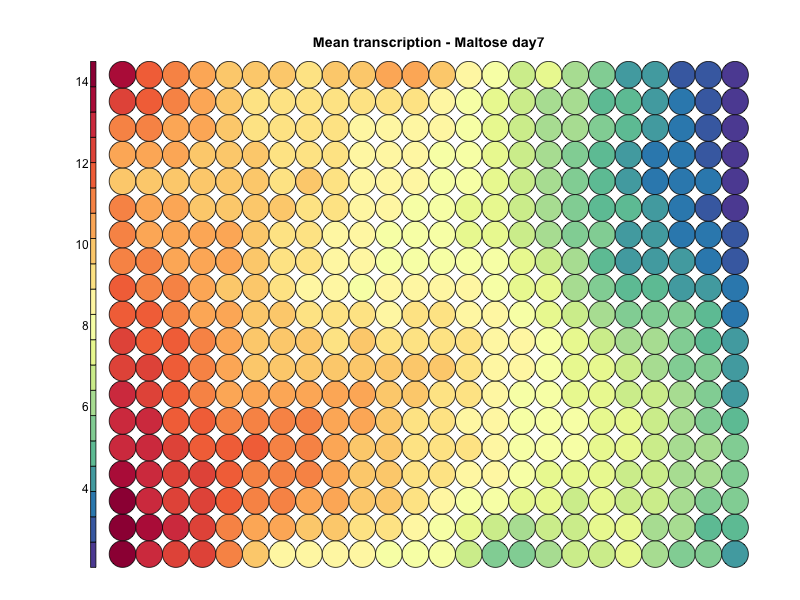

Supplement: S1 File — The animation was made based on the transcriptomic topographies made with mean transcription levels per node for each cultivation condition (Fig 2). (ZIP) [file pone.0175528.s011.zip › S1 File/Pycco310_MeanTranscript_dir/Pycco310_Step10.4_MeanTranscript2.png]

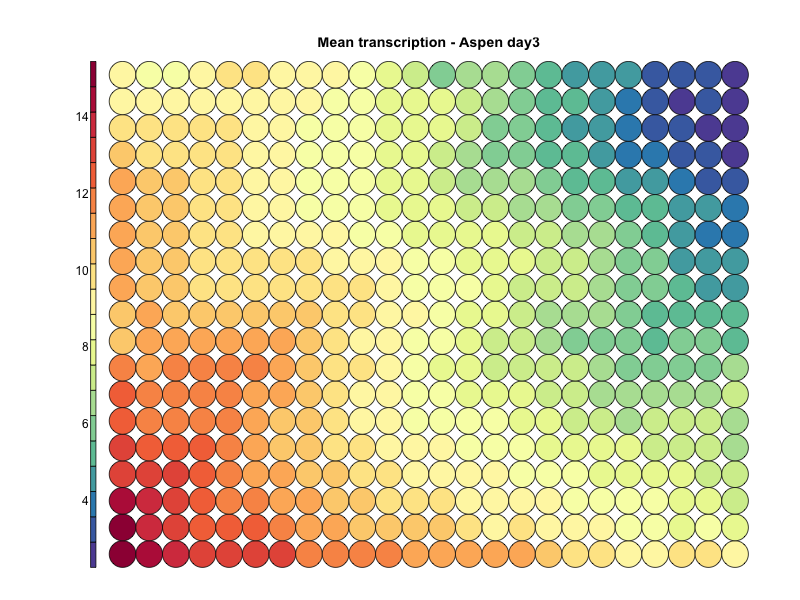

Supplement: S1 File — The animation was made based on the transcriptomic topographies made with mean transcription levels per node for each cultivation condition (Fig 2). (ZIP) [file pone.0175528.s011.zip › S1 File/Pycco310_MeanTranscript_dir/Pycco310_Step10.4_MeanTranscript3.png]

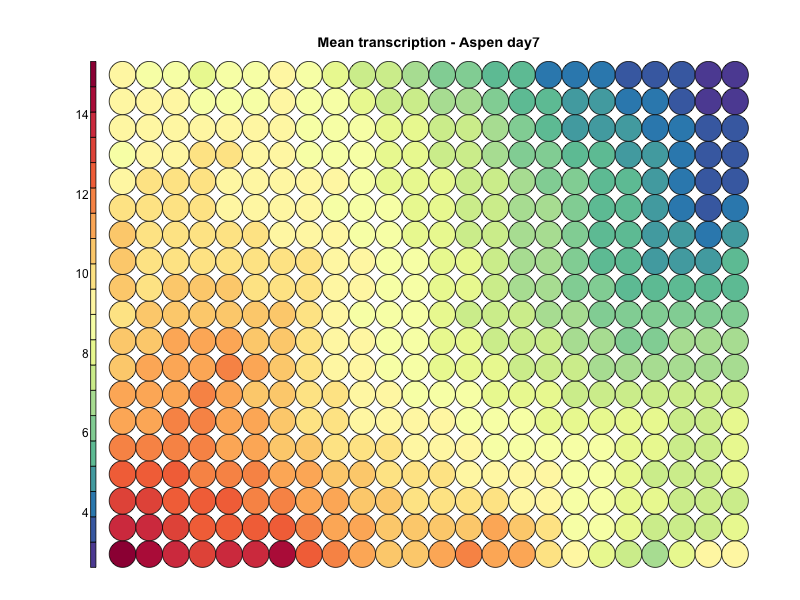

Supplement: S1 File — The animation was made based on the transcriptomic topographies made with mean transcription levels per node for each cultivation condition (Fig 2). (ZIP) [file pone.0175528.s011.zip › S1 File/Pycco310_MeanTranscript_dir/Pycco310_Step10.4_MeanTranscript4.png]

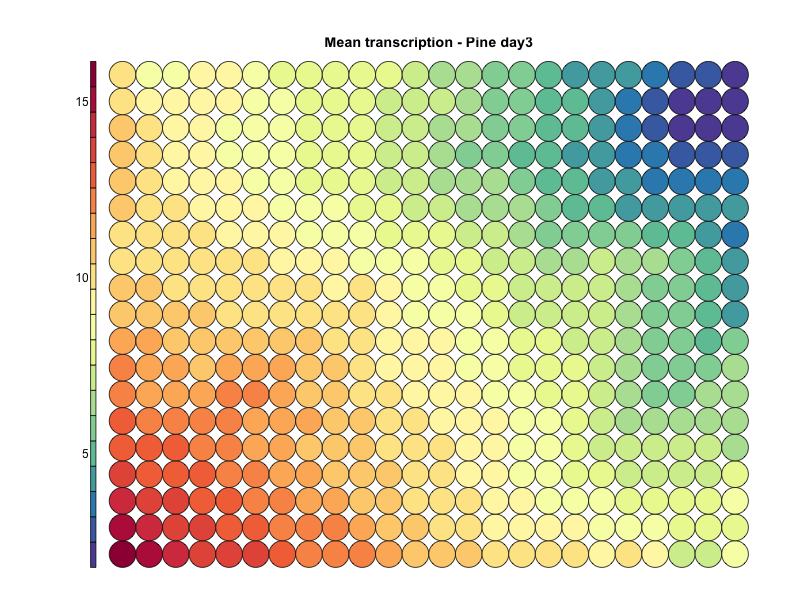

Supplement: S1 File — The animation was made based on the transcriptomic topographies made with mean transcription levels per node for each cultivation condition (Fig 2). (ZIP) [file pone.0175528.s011.zip › S1 File/Pycco310_MeanTranscript_dir/Pycco310_Step10.4_MeanTranscript5.png]

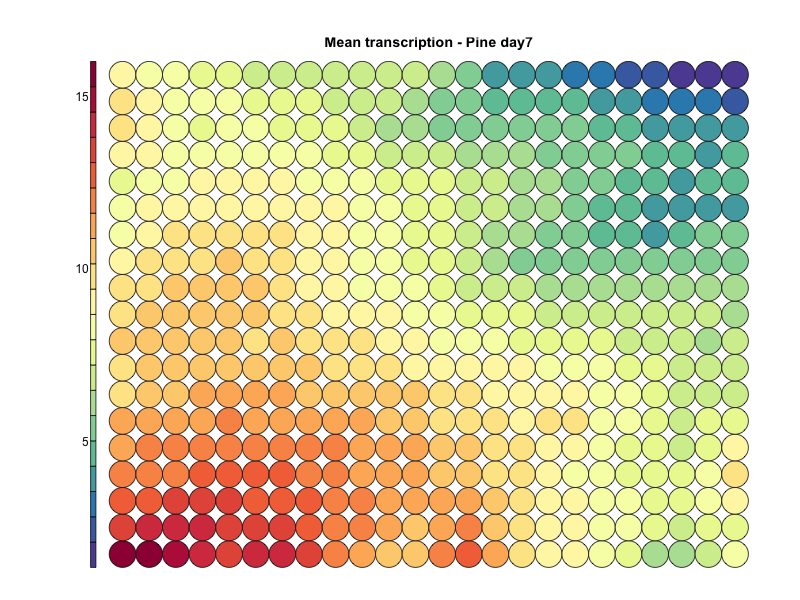

Supplement: S1 File — The animation was made based on the transcriptomic topographies made with mean transcription levels per node for each cultivation condition (Fig 2). (ZIP) [file pone.0175528.s011.zip › S1 File/Pycco310_MeanTranscript_dir/Pycco310_Step10.4_MeanTranscript6.png]

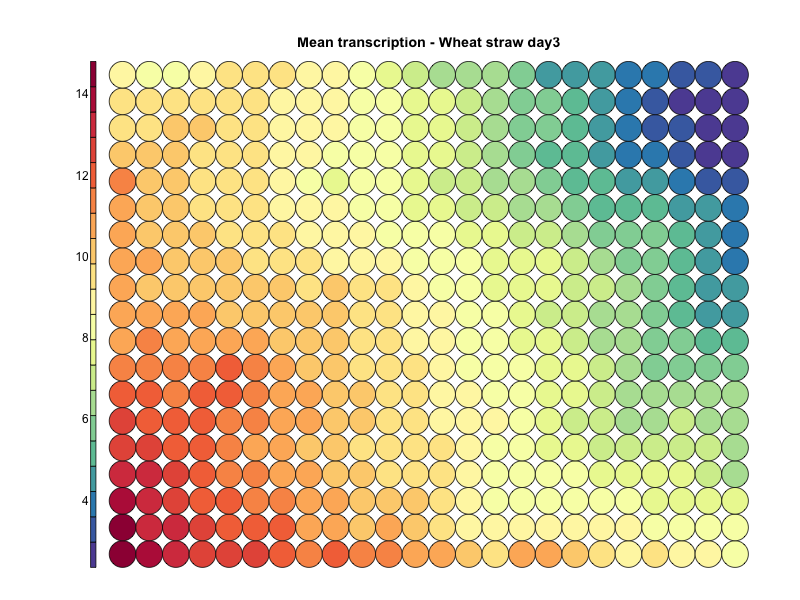

Supplement: S1 File — The animation was made based on the transcriptomic topographies made with mean transcription levels per node for each cultivation condition (Fig 2). (ZIP) [file pone.0175528.s011.zip › S1 File/Pycco310_MeanTranscript_dir/Pycco310_Step10.4_MeanTranscript7.png]

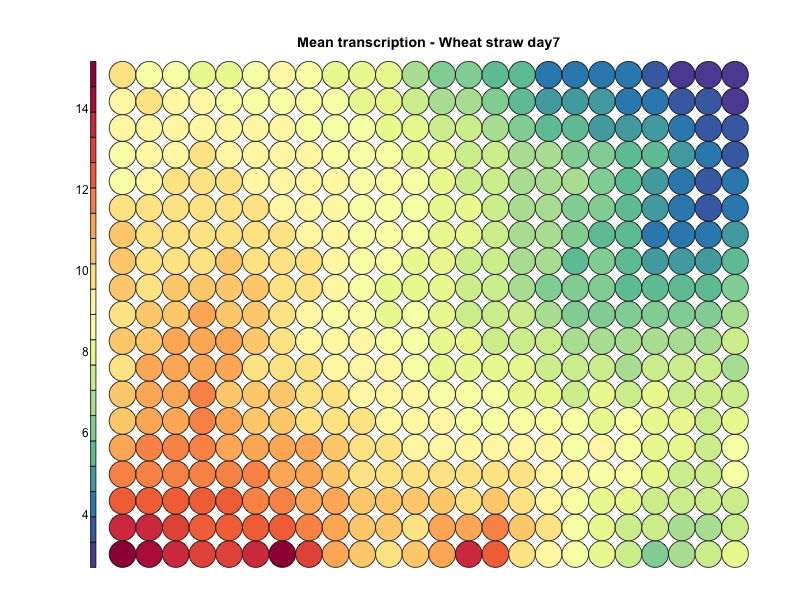

Supplement: S1 File — The animation was made based on the transcriptomic topographies made with mean transcription levels per node for each cultivation condition (Fig 2). (ZIP) [file pone.0175528.s011.zip › S1 File/Pycco310_MeanTranscript_dir/Pycco310_Step10.4_MeanTranscript8.png]
